# Diagnosing flow residuals in coiled cerebral aneurysms by MR angiography: meta-analysis

**DOI:** 10.1007/s00415-013-7053-5

**Published:** 2013-07-28

**Authors:** Jan Menke, Peter Schramm, Jan Martin Sohns, Kai Kallenberg, Wieland Staab

**Affiliations:** 1 Department of Diagnostic Radiology, University Hospital, Robert-Koch-Strasse 40, 37075 Goettingen, Germany; 2 Department of Neuroradiology, University Hospital, Robert-Koch-Strasse 40, 37075 Goettingen, Germany

**Keywords:** Cerebral aneurysm, Coiling, Magnetic resonance angiography, Meta-analysis, Diagnostic accuracy

## Abstract

This meta-analysis summarizes the accuracy of magnetic resonance angiography (MRA) for diagnosing residuals in coiled cerebral aneurysms by using the threefold Roy classification (residuals: none, neck, or sac). Four databases were searched from 2000 to June 2013 for eligible studies that compared MRA to digital subtraction angiography (DSA) and reported 3 × 3 count data of threefold Roy classification, or a reduced scheme of 2 × 2 count data. Bivariate and trivariate Bayesian random-effects models were used for meta-analysis. Among 27 included studies (2,119 coiled aneurysms in 1,809 patients) the average prevalence of DSA-confirmed sac residuals was 18.2 % (range 0–43 %). The pooled sensitivity was 88.0 % (95 % CI 81.4−94.0) and specificity was 97.2 % (94.6−99.0 %) for assessing sac residuals by MRA. In the trivariate meta-analysis, a “sac residual” finding at MRA had a high positive likelihood ratio of 28.2 (14.0–79.0). A “neck residual” finding had a moderate negative likelihood ratio of 0.246 (0.111–0.426), and the MRA finding of “no residual” had a good negative likelihood ratio of 0.044 (0.013–0.096). Subgroup analyses identified no significant influence of covariates on diagnostic accuracy (*P* > 0.05). In conclusion, in coiled cerebral aneurysms MRA with application of the threefold Roy classification is well suited for detecting or excluding sac residuals that might require retreatment.

**Electronic supplementary material:**

The online version of this article (doi:10.1007/s00415-013-7053-5) contains supplementary material, which is available to authorized users.

## Background

Cerebral aneurysms can cause substantial morbidity and mortality, especially if they rupture, leading to non-traumatic subarachnoid haemorrhage [1]. Symptomatic patients are relatively young with an average age of about 50 years, and women are more often affected than men [2, 3]. In unruptured aneurysms the risk of further growth and rupture is individually weighted against potential risks, associated with coiling or clipping [4–8]. Ruptured cerebral aneurysms are often treated by endovascular coiling or neurosurgical clipping to prevent further bleeding [1, 9], with long-term results of coiling being better than with clipping [1, 9, 11]. However, coiling may contain the risk of coil compaction, leading to a potential recurrence of the aneurysm [1, 12]. This occurs in about 20–30 % of cases, half of which are retreated [1, 13]. Coiled aneurysms are therefore followed up routinely by angiography: In case of recanalization of the aneurysm sac, endovascular retreatment is often necessary [14]. In contrast, a pure neck residual is generally not retreated. The threefold Roy classification considers this clinically relevant differentiation between neck and sac residual (class 1 = no residual; class 2 = pure neck residual; class 3 = sac residual) [15, 16].

Follow-up of coiled cerebral aneurysms has been traditionally performed by digital subtraction angiography (DSA), the diagnostic reference standard [1]. Magnetic resonance angiography (MRA) is a noninvasive alternative that can be performed on an outpatient basis, without ionizing radiation, and without catheter-related complications [1, 17]. For this purpose the diagnostic accuracy of MRA must be adequate, which has been investigated by several studies. Most initial studies have performed the “any residual” assessment (Roy class 1 versus classes 2 + 3) that adds neck and sac residuals, although having different clinical consequences. In 2007 and 2008 two meta-analyses summarized those studies [18, 19]. Since then, several new studies were published reporting 3 × 3 count data of the Roy classification. Many added a “sac residual” assessment (Roy classes 1 + 2 versus class 3). In both standard assessments the 3 × 3 count data are reduced to bivariate 2 × 2 count data, causing some loss of information. In particular, this prevents assessing likelihood ratios and predictive values of MRA for the three Roy classes. This requires a trivariate analysis of the 3 × 3 count data, not performed so far.

The purpose of the present meta-analysis was to determine the diagnostic accuracy and predictive value of MRA for assessing flow residuals in coiled cerebral aneurysms compared to DSA, by applying bivariate and trivariate statistical approaches, and by including primary studies that have not been meta-analyzed before.

## Methods

This work applied the Preferred Reporting Items for Systematic reviews and Meta-Analyses (PRISMA guideline) without prepublication of the review protocol [20, 21].

### Data Sources and Searches

The PubMed, Scopus, Biosis and ISI databases were searched for “coiled cerebral aneurysm”, “MRA”, “DSA”, and related terms from January 2000 to June 2013 without language restriction (Online Table e1). Reference lists of retrieved articles were also searched.

### Study selection

Two observers independently selected eligible studies with disagreement solved in consensus. The inclusion criteria were: (a) the patients harbored one or more cerebral aneurysm(s) treated with detachable coils; (b) the study included at least 10 coiled aneurysms and was not limited to specific aneurysm sizes or locations; (c) MRA, the index test, was performed with one or more of these sequences: time-of-flight (TOF-MRA), contrast-enhanced TOF (ceTOF-MRA), and/or contrast-enhanced T1-weighted (CE-MRA); (d) DSA was the reference standard; (e) 3 × 3 count data of the Roy classification, or 2 × 2 count data of the “any residual” or “sac residual” assessment could be reconstructed. A study was excluded if it did not meet all of these inclusion criteria. If a research group reported growing experience in successive publications, then only the most recent publication was included to avoid duplicate counting of findings.

### Data extraction

Data from included studies were independently extracted by two observers using electronic forms, with disagreement solved in consensus. Extracted study characteristics comprised details about study design, patients, MRA, and DSA. Count data were extracted on a per-aneurysm basis. Some additional data were obtained from the study authors via e-mail.

### Study quality and risk of bias

On the study level, the methodological quality and sources of bias were assessed by the 14 quality items of the QUADAS tool [22]. Item 4 was scored positive, if the delay between MRA and DSA was ≤10 days in all patients. For each study a quality score was calculated by assigning 1 point for each QUADAS item if fulfilled, 0.5 points if unclear, and 0 points if not fulfilled. A score ≥11 points was considered as high study quality. On the outcome level, publication bias was assessed by a funnel plot and bivariate meta-regression of the LOR (Logarithm of the diagnostic Odds Ratio) versus the effective sample size parameter, as previously described [23, 24].

### Contingency tables

If any study had investigated more than one MRA sequence, the according raw 2 × 2 or 3 × 3 count data were averaged to obtain one contingency table per study. Such averaging was not performed for subgroup analyses that investigated differences among the MRA sequences.

### Bivariate Meta-analyses

For the “any residual” and “sac residual” assessments, the studies’ sensitivities/specificities as well as Cochran’s *Q*-test and I-squared statistics of between-study heterogeneity were calculated by the freeware program Meta-DiSc [25]. Pooled summary estimates of sensitivity and specificity were obtained from a bivariate random-effects meta-analysis [26, 28]. This kind of meta-analysis models between-study heterogeneity with normally distributed bivariate random effects that account for a possible correlation between sensitivity and specificity [26, 29–31]. The summary estimates were also transformed to positive and negative likelihood ratios that can be used for calculating conditional probabilities (Table e-2) [24, 32–34]. Positive likelihood ratios >10 were considered suitable for confirming aneurysm residuals, and negative likelihood ratios <0.1 suitable for excluding them.

### Trivariate meta-analysis

However, the bivariate standard assessments do not fully consider the threefold Roy classification, since they add neck residuals to either the “no residual” or “sac residual” class. Therefore, an additional trivariate random-effects meta-analysis was performed with 3 × 3 count data of the Roy classification [26, 31, 35]. In this approach three likelihood ratios were derived. They indicate the relative likelihood of having a DSA-confirmed sac residual, if MRA indicates “no residual” (first negative likelihood ratio, LRN_1_), or if MRA indicates a “neck residual” (second negative likelihood ratio, LRN_2_), or if MRA indicates a “sac residual” (positive likelihood ratio, LRP). These three likelihood ratios were used for generating a trivariate graph of conditional probabilities (Table e-3).

### Subgroup analyses

Potential sources of heterogeneity were assessed by subgroup analyses of the “sac residual” assessment, with the study characteristics as categorical covariates in bivariate random-effects meta-regressions [36]. In each subgroup the overall diagnostic accuracy was indicated by the LOR, with LOR = logit(sensitivity) + logit(specificity) [31, 37]. A significant difference between the subgroups was assessed, if the 95 % credible interval (95 % CI) of the LOR-difference excluded zero (*P* < 0.05).

### Bayesian meta-analysis program

Meta-analyses were performed with the Bayesian PROC MCMC from SAS 9.3 (SAS Institute Inc., Cary, NC, USA) that obtains its results from numerical simulation rather than analytically or by iterative approximation [26–28]. Referring to the Bayesian reporting guideline “ROBUST” [38], the program has the following characteristics: A multivariate Normal prior with large variances was used for the logit-transformed pooled sensitivity and specificity. In the relevant logit-range of −10 to 10, this prior is nearly uniform (flat), and thus uninformative. This logit-range corresponds to a sufficiently broad range of 0.005–99.995 % for sensitivity and specificity on the probability scale. For the random effects a bivariate Normal prior was used [26–31]. A sensitivity analysis was performed by using different initials for the priors [26]. The program output included the mean (measures the central tendency) and 95 % credible interval (95 % CI measures the variability) of the model estimates [26]. The latter corresponds to a “95 % confidence interval” in classical statistics. The trivariate meta-analysis is an extension of the bivariate meta-analysis [26, 31, 35]. The meta-analytic programs always converged to unique posterior distributions.

## Results

### Literature search and selection

**Fig. 1:**
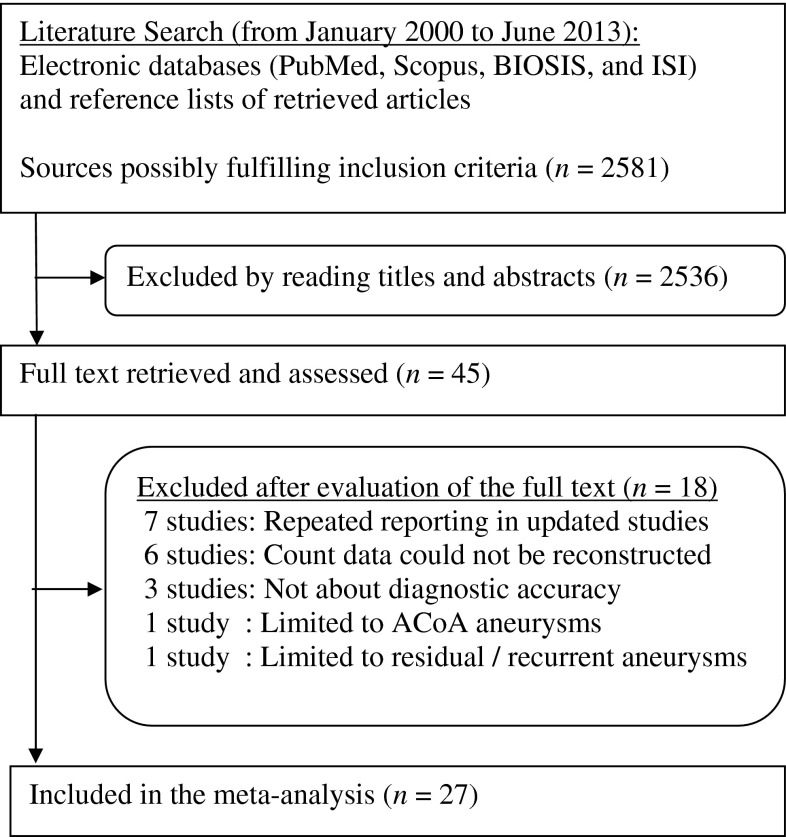
Study flow chart

Among 2,581 retrieved sources, 2,536 were excluded by reading titles and abstracts, and 18 by evaluating the full text (Fig. 1). The remaining 27 studies were included. While 10 studies [e1−e10] had been part of two previous meta-analyses [17–19], the remaining studies had not been meta-analyzed before [e11−e27].

### Descriptive statistics

Nineteen studies were prospective, six retrospective, and in two studies the design was unclear. One multicenter study provided data from four centers [e23]. On average, about 69 % of patients were female (range 36–92 %), the mean/median patient age was 51 years (range 8–90 years), and about 85 % of patients had received coiling of a ruptured aneurysm (range 5–100 %). Most patients had a single coiled aneurysm (mean 1.2 coiled aneurysms per patient). The 27 studies had included 2,119 coiled aneurysms of 1,809 patients. About 80 % of the aneurysms were located in the anterior and 20 % in the posterior circulation [e1−e27]. Twelve studies reported the aneurysm size before coiling in millimeter categories [e1, e2, e4, e5, e7−e11, e18, e24, e27]. About 29 % of them were small (up to 4 mm), 46 % medium-sized (5–10 mm), and 25 % large (≥11 mm). Six studies reported 2 × 2 count data exclusively of the “any residual” assessment (Roy class 1 versus classes 2 + 3) in 566 aneurysms. The other 21 studies provided 3 × 3 count data in 1,553 aneurysms. Among these 21 studies, DSA showed on average 53.8 % complete occlusions (range 14–91 %), 28.0 % neck residuals (range 4–68 %), and sac residuals 18.2 % (range 0–43 %). In most cases, follow-up was performed 3–24 months after coiling. Twelve studies compared two or more different MRA sequences to DSA. In total, 1,365 coiled aneurysms were studied by 1,216 TOF-MRA, 232 aneurysms by 206 ceTOF-MRA, 660 aneurysms by 569 CE-MRA, and 503 aneurysms by 460 MRA with mixed sequences. About 72 % of MRA were performed at 1.5 Tesla and 28 % at 3.0 Tesla. In most studies the MRA readers were blinded to the DSA results, and vice versa (eFig. 1). Further descriptive data are provided in Table e-4 (study characteristics) and Table e-5 (reasons for exclusion of patients/aneurysms from the primary studies).

### Study quality, publication bias, and heterogeneity

The study quality was generally high (eFig. 1). The funnel plot and regression test indicated no significant publication bias (*P* = 0.44). The between-study heterogeneity was low to moderate (*P* < 0.05;* I* ^2^ 29–72 %).

### Reproducibility of MRA

In 10 studies the interobserver reproducibility of MRA was reported by κ—statistics [e10, e16, e18−e20, e22−e24, e26, e27]. The median κ-statistics was 0.60 (range 0.56–0.80) for the bivariate “any residual” assessment, 0.74 (0.63–0.77) for the bivariate “sac residual” assessment, and 0.64 (0.49–0.93) for the trivariate assessment, indicating moderate to good inter-observer agreement. Five of these studies compared TOF-MRA versus CE-MRA and/or compared MRA at 1.5 versus 3.0 Tesla [e20, e22, e24, e26, e27]. Among these studies, the according interobserver reproducibility showed no consistent trend in favor of a certain MRA technique.

### Count data of individual studies

For the individual studies, 2 × 2 count data of the “any residual” and “sac residual” assessment and corresponding estimates of sensitivity and specificity are given in Table e-6. Table e-7 provides trivariate 3 × 3 count data of the Roy classification.

### Bivariate meta-analysis of sensitivity and specificity

Diagnosing “any residual” (neck or sac) by MRA showed a pooled sensitivity of 89.0 % (95 % CI, 85.1−92.6 %) and specificity of 89.0 % (82.6−94.0 %) (Table 1). Diagnosing a “sac residual” had a pooled sensitivity of 88.0 % (95 % CI, 81.4−94.0 %) and specificity of 97.2 % (94.6−99.0 %). The positive likelihood ratio was high for diagnosing a “sac residual” (mean 33.6) and moderate for diagnosing “any residual” (mean 8.4). Both standard assessments showed moderate negative likelihood ratios (mean 0.12).

**Table 1:** Bivariate meta-analytic summary estimates

Meta-analytic parameters	Aneurysm assessment	
	Any residual^a^	Sac residual^b^
Study centers	30	23
Coiled aneurysms	2,119	1,553
Sensitivity (95 % CI), %	89.0 (85.1–92.6)	88.0 (81.4–94.0)
Specificity (95 % CI), %	89.0 (82.6–94.0)	97.2 (94.6–99.0)
LOR (95 % CI)	4.22 (3.57–4.95)	5.65 (4.56–7.00)
LRP (95 % CI)	8.35 (5.10–14.87)	33.6 (15.7–89.5)
LRN (95 % CI)	0.12 (0.08–0.17)	0.12 (0.06–−0.19)

LOR, logarithm of the diagnostic odds ratio

LRP positive likelihood ratio

LRN negative likelihood ratio

^a^ No residual (class 1) versus neck or sac residual (classes 2 or 3)

^b^ No or neck residual (classes 1 or 2) versus sac residual (class 3)

### Trivariate meta-analysis and conditional probabilities

**Fig. 2:**
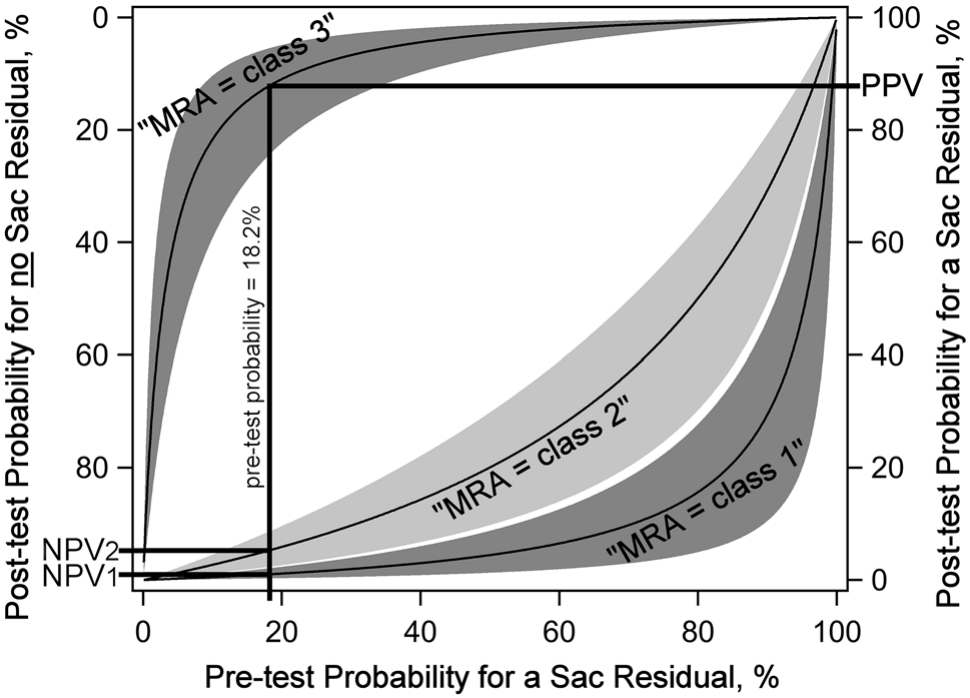
Trivariate conditional probabilities. This graph shows post-test probabilities for having or not having an aneurysmal sac residual, depending on the pre-test probability for sac residuals, and depending on the test result of MRA. This trivariate MRA result is categorized as Roy class 1 (no residual), class 2 (neck residual), or class 3 (sac residual). The central black curves are means, and the surrounding wings represent the according 95 % CI. An example is given for the studies’ average prevalence of sac residuals that was 18.2 % (*vertical line*, indicated by “pre-test probability = 18.2 %”). If MRA indicates a “class 1 = no residual” finding, then the average post-test probability for truly having no sac residual is 99.0 %, which is the studies’ mean negative predictive value 1 (NPV_1_). If MRA indicates a “class 2 = neck residual” finding, then the average post-test probability for truly having no sac residual is 94.7 %, which is the studies’ mean negative predictive value 2 (NPV_2_). If MRA indicates a “class 3 = sac residual” finding, then the average post-test probability for truly having a sac residual is 87.9 %, which is the studies’ mean positive predictive value (PPV)

The Roy class of aneurysm residuals was correctly assessed by MRA in 86.8 % (95 % CI, 80.5–91.8 %) of cases, whereas underestimation occurred in 5.6 % (3.5–8.0 %) and overestimation in 7.6 % (3.7–12.9 %) of cases. It was rare that MRA deviated from DSA by two classes, i.e. that MRA indicated “no residual” in a DSA-confirmed sac residual (2.7 %) or a “sac residual” in a DSA-confirmed occluded aneurysm (1.7 %). The positive likelihood ratio (LRP = 28.2) of “sac residual at MRA” was above 10, indicating the suitability of MRA for detecting true sac residuals. The negative likelihood ratio of “no residual at MRA” (LRN_1_ = 0.044) was below 0.1, indicating this finding’s suitability for excluding sac residuals. The negative likelihood ratio of “neck residual at MRA” (LRN_2_ = 0.246) was above 0.1, indicating limited value of that intermediate finding for excluding sac residuals. Corresponding trivariate conditional probabilities are presented in Fig. 2. At a pretest probability of 18.2 % for sac residuals (the average prevalence among the studies), a “neck residual” finding at MRA has a negative predictive value (NPV_2_) of about 94.7 %, and is therefore still a useful finding (Table 2). At that pretest probability, a “no residual at MRA” finding excludes a sac residual in about 99.0 % of cases, and a “sac residual at MRA” finding is true in about 87.9 % of cases.

**Table 2:** Trivariate meta-analytic summary estimates

	*DSA*			Likelihood ratios	Predictive values
	No residual	Neck residual	Sac residual		
	(Roy class 1)	(Roy class 2)	(Roy class 3)	For sac residuals	For sac residuals^a^
*MRA*					
No residual	**89.0** **%**	*12.9* *%*	*2.7* *%*	LRN_1_ = 0.044	NPV_1_ = 99.0 %
(Roy class 1)	**(80.6–95.1** **%)**	*(6.9–19.8* *%)*	*(0.8–5.6* *%)*	(0.013–0.096)	(97.9–99.7 %)
*MRA*					
Neck residual	9.3 %	**81.0** **%**	*8.6* *%*	LRN_2_ = 0.246	NPV_2_ = 94.7 %
(Roy class 2)	(4.0–16.6 %)	**(71.4–89.4** **%)**	*(3.7−14.3* *%)*	(0.111–0.426)	(91.4–97.6 %)
*MRA*					
Sac residual	1.7 %	6.1 %	**88.7** **%**	LRP = 28.2	PPV = 87.9 %
(Roy class 3)	(0.5–3.5 %)	(2.0–11.3 %)	**(82.7–94.2** **%)**	(14.0–79.0)	(75.6–94.5 %)
Sum	100 %	100 %	100 %		

^a^At an average pretest probability of 18.8 % for the prevalence of sac residuals

*LRN*_*1*_ negative likelihood ratio 1 (for sac residual at DSA, if MRA indicates no residual), *LRN*_*2*_ negative likelihood ratio 2 (for sac residual at DSA, if MRA indicates a neck residual), *LRP* positive likelihood ratio (for sac residual at DSA, if MRA indicates a sac residual), *NPV*_*1*_ negative predictive value 1 (for excluding a true sac residual, if MRA indicates no residual), *NPV*_*2*_ negative predictive value 2 (for excluding a true sac residual, if MRA indicates a neck residual), *PPV* positive predictive value (for detecting a true sac residual, if MRA indicates a sac residual)

The 3 × 3 count data from 21 studies (1,553 coiled aneurysms) were summarized by a random-effects meta-analysis. The meta-analytic results are expressed as probabilities (with 95 % CI in *brackets*). With DSA as reference standard, MRA correctly assessed the residual status in 86.7 % (95 % CI: 80.5–91.8) of coiled aneurysms (bold, diagonal), underestimated 5.6 % (3.5–8.0 %) of residuals (*italic, upper right triangle*), and overestimated residuals in 7.6 % (3.7−12.9 %) of cases (*lower left triangle*). Both right columns present likelihood ratios (LR) and predictive values (PV) for sac residuals. The predictive values were estimated for a pretest probability of 18.2 %, which was the studies’ average prevalence of sac residuals

### Meta-analytic subgroup analyses

The subgroup analyses showed no significant differences in diagnostic accuracy (*P* > 0.05). This referred to MRA sequence (TOF-MRA versus CE-MRA), field strength (1.5 versus 3.0 Tesla), number of MRA readers (1 versus 2–3), and several other covariates (Table e-8). The 19 included prospective studies showed results similar to those reported in Tables 1 and 2.

## Discussion

This meta-analysis confirms that noninvasive MRA is generally well suited for assessing flow residuals in coiled cerebral aneurysms. This is relevant both to imaging specialists and referring clinicians, since both partners decide about using DSA or MRA for the follow-up of the coiled aneurysms, and about how to proceed with the obtained imaging results. MRA is attractive, since it can be performed on an outpatient basis, is fast, obviates catheter-related complications, and requires less personnel than DSA. This is the first meta-analysis that fully considers the threefold Roy classification of aneurysm residuals (no, neck, or sac residual) [15, 16]. It shows that the according threefold MRA results have different likelihoods for detecting/excluding a true sac residual (that might require retreatment). This should be considered in MRA-based decision making.

The reported per-aneurysm results approximately represent per-patient results, since most patients had a single coiled aneurysm. DSA showed on average 53.8 % complete occlusions (Roy class 1), 28.0 % neck residuals (class 2), and 18.2 % sac residuals (class 3). MRA correctly assessed about 86.8 % of cases, and over-/underestimated 8.8 % of cases by one Roy class. Over-/underestimation by two classes was more rare (4.4 %).

Differentiating neck and sac residuals is clinically relevant, since about 50 % of sac residuals are retreated, whereas neck residuals are generally not retreated [1, 10]. Neck residuals and no residuals therefore generally have the same consequence of watchful waiting. Their MRA-based differentiation is nevertheless useful, since “no residual at MRA” rules out a sac residual with higher diagnostic confidence than “neck residual at MRA”.

The simple bivariate “any residual” assessment (no residual versus any neck/sac residual) might indicate just moderate diagnostic accuracy of MRA for aneurysm residuals. However, in that assessment the sac and neck residuals are summed to one group, although having different therapeutic implications. The according statistical results are therefore clinically less meaningful than the bivariate “sac residual” assessment or the more detailed trivariate assessment.

The refined bivariate “sac residual” assessment (no or neck residual versus sac residual) shows a high specificity, i.e. the number of false-positive sac residual findings at MRA is small. Accordingly, the positive predictive value is nearly similar to the trivariate assessment. However, the bivariate “sac residual” assessment does not differentiate the predictive value of the Roy classes 1 and 2.

The trivariate meta-analytic assessment is considered to be the best approach, since it fully accounts for the 3×3 data of the Roy classification. In the trivariate meta-analysis, the finding of “no residual at MRA” showed a good negative likelihood ratio (mean 0.044) for ruling out a true sac residual. Among the studies the mean prevalence of sac residuals was 18.2 %, and at this prevalence an MRA finding of “no residual” truly excludes a sac residual in 99.0 % of cases, obviating the need for additional DSA. In aneurysms with adequate occlusion 6 months after coiling (those <10 mm and not located on the basilar tip), prolonged imaging follow-up seems unnecessary, since late reopening with need of retreatment is very rare [39].

The intermediate finding of “neck residual at MRA” had only a moderate negative likelihood ratio (mean 0.246) for ruling out a true sac residual. However, the according predictive value depends on the pretest probability, as visualized in the graph of conditional probabilities (Fig. 2). At a pretest probability of 18.2 % for sac residuals, this MRA finding is still useful, since the associated negative predictive value is high (mean 94.7 %). Additional DSA may be considered optional, since it shows a sac residual in only about 5.3 % of cases, and therefore further follow-up is a suitable diagnostic alternative.

The finding of “sac residual at MRA” had a high positive likelihood ratio (mean 28.2), and is therefore well suited for detecting a true sac residual. DSA may then be subsequently performed to exclude some false-positives, and to evaluate retreatment in the approximately 87.9 % of cases with true sac residuals [1, 10]. However, such DSA is optional on an individual basis, since MRA may generally be used for deciding about retreatment versus further follow-up [40]. Most studies of this meta-analysis were prospective with consecutive patient enrollment and blinding of MRA versus DSA assessments, contributing to class I evidence that MRA is useful for diagnosing flow residuals in coiled cerebral aneurysms.

Some primary studies had found differences in diagnostic accuracy among different MRA methodologies. However, on the meta-analytic level the according subgroup analyses showed no consistent significant differences. For example, MRAs with 1.5 Tesla magnets generally performed similar to 3.0 Tesla. Additionally, double-reading of MRA is not mandatory, since single-reading has similar diagnostic accuracy, if performed by an appropriately trained radiologist. The accuracy of TOF-MRA was not significantly different from contrast-enhanced MRA. While the former requires more acquisition time than the latter, it saves on the costs of contrast medium and avoids the small potential risks of contrast medium in patients with renal failure.

Two previous meta-analyses summarized findings in about 800–880 coiled aneurysms from studies published between 1997 and 2006 [18, 19]. Our meta-analysis applied a generalized linear mixed model that is considered more appropriate than the previously applied general model [18, 19, 26, 31]. The primary studies of both previous meta-analyses had provided count data only for the 2×2 “any residual” assessment. Since then several studies have been published providing 3 × 3 count data of the Roy classification. Therefore, the current meta-analysis includes 17 novel studies, increases the number of meta-analysed aneurysms to more than 2,100, and can provide the “sac residual” and trivariate assessments.

This meta-analysis has some limitations. MRA after stent-assisted coiling was not meta-analysed, since according publications are currently too few [41–44]. This meta-analysis did not stratify the diagnostic accuracy of MRA for aneurysm size because of having no data. According to two primary studies, the sensitivity and specificity of MRA for diagnosing sac residuals after coiling is lower in small aneurysms than in large aneurysms [e11, e25]. Consequently, the diagnostic accuracy of MRA in large aneurysms would be higher than indicated by our pooled estimates, which also summarize small aneurysms. This is favorable, since coiled large aneurysms require retreatment more frequently than small aneurysms [14].

In conclusion, non-invasive MRA with application of the threefold Roy classification is well suited for assessing flow residuals in coiled cerebral aneurysms. A “sac residual” finding at MRA should be confirmed by catheter angiography to exclude false-positives and to possibly retreat the true-positives. A “neck residual” finding at MRA should be followed up further. An MRA finding of “no residual” generally requires no confirmation by DSA.

## Electronic supplementary material

Below is the link to the electronic supplementary material.

Supplementary material 1 (PDF 270 kb)

## Abbreviations

DSA: Digital subtraction angiography
MRA: Magnetic resonance angiography
CE-MRA: Contrast-enhanced T1-weighted MRA
TOF-MRA: Time-of-flight MRA
ceTOF-MRA: Contrast-enhanced TOF-MRA
