# Supplementary material for: Diagnosing flow residuals in coiled cerebral aneurysms by MR angiography: meta-analysis
Source: J Neurol. 2013 Jul 28;261(4):655–62. doi: 10.1007/s00415-013-7053-5 (PMC3973941; doi:10.1007/s00415-013-7053-5)
Supplement: Supplementary file 1 — Supplementary material 1 (PDF 270 kb) [file 415_2013_7053_MOESM1_ESM.pdf]

SUPPLEMENT: Table of Content

	Supplemental Material	Page
**eFig. 1**	**Summary of Study Quality**	2
**eTable 1**	**Literature Search**	3
**eTable 2**	**Bivariate Likelihood Ratios and Conditional Probabilities**	
	A. Estimation of bivariate post-test probabilities	4
	B. Bivariate likelihood ratios for assessing any residuals by MRA	4
	C. Bivariate likelihood ratios for assessing sac residuals by MRA	4
**eTable 3**	**Trivariate Likelihood Ratios and Conditional Probabilities**	
	A. Estimation of trivariate post-test probabilities	5
	B. Trivariate likelihood ratios for assessing sac residuals by MRA	5
**eTable 4**	**Study Characteristics**	
	A. Study Design	6
	B. Patients and Coiled Aneurysms	7–8
	C. Index Test (MRA)	9–12
	D. Reference Test (DSA)	13
**eTable 5**	**Excluded Patients and Aneurysms**	
	A. Exclusion of Patients from the Studies	14–15
	B. Exclusion of MRAs and Aneurysms from Analysis	16
**eTable 6**	**Bivariate Count Data of MRA versus DSA**	
	A. Bivariate Assessment of Any Residuals	17–18
	B. Bivariate Assessment of Sac Residuals	19–20
**eTable 7**	**Trivariate Count Data of MRA versus DSA**	21–22
**eTable 8**	**Subgroup Analyses with Study Characteristics as Covariates**	23–24
**eTable 9**	**Supplemental References of the Primary Studies**	25–26

eFig. 1 Summary of Study Quality

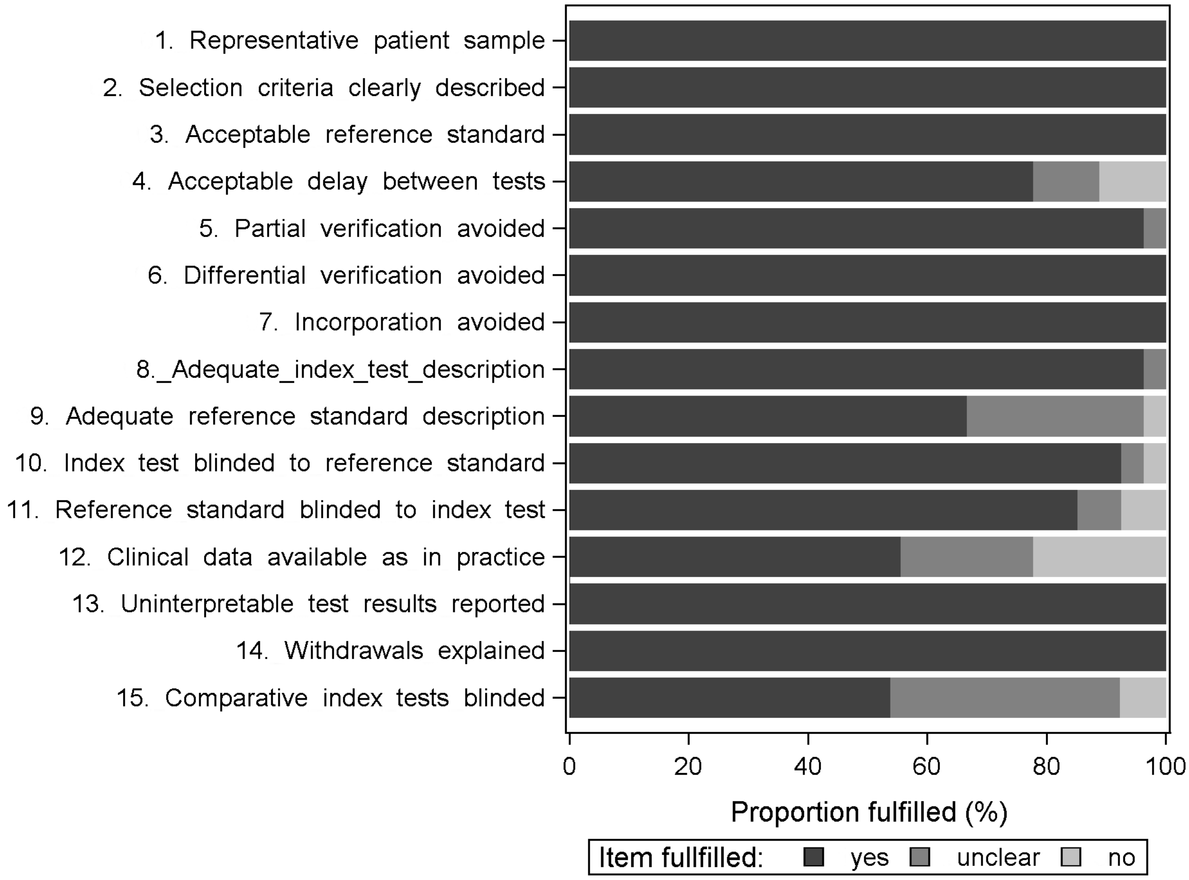

The study quality was assessed by 15 items (see Methods). The consensus judgment of two readers is shown as cumulative percentages across the 27 primary studies.

eTable 1. Literature Search of the PubMed Database

No.	Search Terms *
Patient's Condition	
#1.	aneurysm*
#2.	coil*
#3.	#1 and #2
Index Test	
#4.	magnetic resonance angiography
#5.	MR angiography
#6.	MRA
#7.	MR angiogram
#8.	MR angiographic
#9.	#4 or #5 or #6 or #7 or #8
Reference Method	
#10.	digital subtraction angiography
#11.	DSA
#12.	IADSA
#13.	angiography
#14.	angiographic
#15.	angiogram
#16.	#10 or #11 or #12 or #13 or #14 or #15
Outcome Measure	
	(no limiting filters applied)
Combination of Search Terms	
#17.	#3 and #9 and #16
#18.	#17 2000:2013 [DP]

* Last searched on July 10, 2013

eTable 2. Bivariate Likelihood Ratios and Conditional Probabilities

eTable 2A. Estimation of bivariate post-test probabilities

Symbol	Parameter name	Formula
p11	specificity (true-negative rate)	p11 = specificity
p12	false-positive rate	p12 = 1 – specificity
p21	false-negative rate	p21 = 1 – specificity
p22	sensitivity (true-positive rate)	p22 = sensitivity
LRN	negative likelihood ratio	see tables B and C below
LRP	positive likelihood ratio	see tables B and C below
preP	pre-test probability	can range from 0% to 100%
preOR	pre-test odds ratio	preOR = preP / (1 – preP)
NpostOR	negative post-test odds ratio	NpostOR = preOR * LRN
PpostOR	positive post-test odds ratio	PpostOR = preOR * LRP
NpostP	negative post-test probability	NpostP = 1 – (NpostOR / (1 + NpostOR))
PpostP	positive post-test probability	PpostP = PpostOR / (1 + PpostOR)

eTable 2B. Bivariate likelihood ratios for assessing any residuals by MRA*

		DSA: No residual	***DSA:*** Neck residual or Sac residual	***Likelihood Ratios***
MRA:	No residual	p11	p21	LRN = p21 / p11
MRA:	Neck residual or Sac residual	p12	p22	LRP = p22 / p12
		sum = 100%	sum = 100%	

eTable 2C. Bivariate likelihood ratios for assessing sac residuals by MRA*

		DSA: No residual or Neck residual	***DSA:*** Sac residual	***Likelihood Ratios***
MRA:	No residual or Neck residual	p11	p21	LRN = p21 / p11
MRA:	Sac residual	p12	p22	LRP = p22 / p12
		sum = 100%	sum = 100%	

* In Tables B and C the probabilities "p" are estimated from 2×2 count data by meta-analysis. The bivariate likelihood ratios LRN and LRP are derived from these probabilities.

eTable 3. Trivariate Likelihood Ratios and Conditional Probabilities

The trivariate conditional probabilities are derived analogous to the bivariate conditional probabilities, which are described in eTable 2.

eTable 3A. Estimation of trivariate post-test probabilities

Symbol	Parameter name	Formula
LRN_1	negative likelihood ratio 1	see table below
LRN_2	negative likelihood ratio 2	see table below
LRP	positive likelihood ratio	see table below
preP	pre-test probability	can range from 0% to 100%
preOR	pre-test odds ratio	preOR = preP / (1 − preP)
$NpostOR_1$	negative post-test odds ratio 1	$NpostOR_1 = preOR * LRN_1$
$NpostOR_2$	negative post-test odds ratio 2	$NpostOR_2 = preOR * LRN_2$
PpostOR	positive post-test odds ratio	PpostOR = preOR * LRP
$NpostP_1$	negative post-test probability 1	$NpostP_1 = 1 - (NpostOR_1 / (1 + NpostOR_1))$
$NpostP_2$	negative post-test probability 2	$NpostP_2 = 1 - (NpostOR_2 / (1 + NpostOR_2))$
PpostP	positive post-test probability	PpostP = PpostOR / (1 + PpostOR)

eTable 3B. Trivariate likelihood ratios for assessing sac residuals by MRA

	DSA:<br>No residual or Neck residual	***DSA:***<br>Sac residual	***Likelihood Ratios***
MRA: No residual	p11	p21	LRN_1 = p21 / p11
MRA: Neck residual	p12	p22	LRN_2 = p22 / p12
MRA: Sac residual	p13	p23	LRP = p23 / p13
	sum = 100%	sum = 100%	

The 3×3 count data of the primary studies are reduced to 2×3 count data, as shown.
The probabilities "p" are estimated from the 2×3 count data by meta-analysis.
The trivariate likelihood ratios are derived from these probabilities.

eTable 4. Characteristics of Included Studies

eTable 4A. Study Design

Ref.	Authors, year	Prospec-tive study design	Conse-cutive patients	Study period from–to, *month/year*	Study dura-tion, *months*	Time from coiling to follow-up, mean or *median (range), *months*	Delay between DSA and MRA, mean (max), *days*
e1	Anzalone, 2000	NA	NA	NA	NA	10* (0–36)	NA (2)
e2	Boulin, 2001	Yes	Yes	11/98–06/99	8	12* (3– >24))	1 (1)
e3	Michardiere, 2001	Yes	NA	01/95–08/99	56	17 (2–60)	16 (NA)
e4	Cottier, 2003	Yes	NA	NA	NA	12* (3–48)	NA (7)
e5	Okahara, 2004	Yes	Yes	02/00–05/02	27	6 (0–24)	NA (5)
e6	Yamada, 2004	No	Yes	02/02–10/03	20	9 (5–41)	NA (7)
e7	Farb, 2005	NA	NA	NA	18	6* (0–18)	11 (58)
e8	Majoie, 2005	Yes	Yes	11/03–07/04	9	6 (4–14)	0-1 (1)
e9	Westerlaan, 2005	No	Yes	01/98–01/02	49	3* (3–18)	NA (3)
e10	Pierot, 2006	Yes	Yes	NA	NA	12* (12– >36)	NA (2)
e11	Deutschmann, 2007	No	Yes	10/00–11/02	26	NA (0–59)	NA (2)
e12	Wong, 2007	Yes	Yes	10/03–09/06	36	11 (0–66)	22 (114)
e13	Gauvrit, 2008	No	Yes	01/98–08/01	44	13 (5–25)	5 (NA)
e14	Lubicz, 2008	No	Yes	05/06–05/07	13	12* (6–12)	NA (10)
e15	Ramgren, 2008	Yes	Yes	03/04–04/05	14	27* (3–84)	0-1 (39)
e16	Urbach, 2008	Yes	Yes	NA	NA	14 (0–24)	NA (NA)
e17	Wikstrom, 2008	Yes	No	NA	NA	14* (5–87)	0 (0)
e18	Buhk, 2009	Yes	Yes	11/05–02/07	16	6 (6–6)	0-1 (1)
e19	Ferre, 2009	Yes	Yes	04/04–11/06	31	8 (2–54)	NA (2)
e20	Kau, 2009	No	Yes	12/06–02/08	15	12* (3–19)	NA (8)
e21	Bakker, 2010	Yes	Yes	04/04–03/09	60	3 (3–3)	0 (0)
e22	Kaufmann, 2010	Yes	Yes	NA	36	12 (5–72)	1* (8)
e23	Schaafsma, 2010	Yes	Yes	05/05–11/07	31	6 (3–18)	0 (0)
e24	Nakiri, 2011	Yes	Yes	11/09–05/10	7	NA (6–36)	0-1 (1)
e25	Lavoie, 2012	Yes	Yes#	10/05–05/07	19	12* (6– >20)	0 (0)
e26	Pierot, 2012	Yes	Yes	04/06–09/08	30	12* (3–215)	0-1 (1)
e27	Pierot, 2012	Yes	Yes	04/06–09/08	30	12* (3–215)	0-1 (1)

* median
# with random selection
MRA, magnetic resonance angiography
NA, not available

eTable 4B. Patients and Coiled Aneurysms

			Patients					MRA and aneurysms	
Ref.	Authors, year	MRA sequence (and field strength in *Tesla*)	Patients excluded from study, *n*	Patients (and coiled aneurysms) included in study, *n (n)*	Percentage of women, *%*	Age, mean or *median (range), *years*	Patients with ruptured aneurysms, *%*	Aneurysms (and MRA) excluded from analysis, *n*	Aneurysms (and MRA) included in the analysis, *n*
e1	Anzalone et al., 2000	TOF-MRA (1.5)	0	49 (50)	69	53 (*NA*)	NA	7 (7)	57 (56)
e1	Anzalone et al., 2000	ceTOF-MRA (1.5)	25	24 (25)	69	53 (*NA*)	NA	4 (4)	21 (20)
e2	Boulin et al., 2001	ceTOF-MRA (1.5)	2	66 (70)	55	45 (*NA*)	79	1 (1)	80 (80)
e3	Michardiere et al., 2001	TOF-MRA (1.5)	0	20 (22)	65	48 (37–69)	90	0 (0)	25 (23)
e4	Cottier et al., 2003	TOF-MRA (1.5)	0	58 (71)	81	52 (*NA*)	84	1 (1)	70 (57)
e4	Cottier et al., 2003	ceTOF-MRA (1.5)	0	58 (71)	81	52 (*NA*)	84	1 (1)	70 (57)
e5	Okahara et al., 2004	TOF-MRA (1.5)	0	33 (33)	70	63 (31–84)	67	3 (3)	30 (30)
e6	Yamada et al., 2004	TOF-MRA (1.5)	31	39 (39)	77	59 (39–76)	5	0 (0)	51 (51)
e7	Farb et al., 2005	TOF-MRA (1.5)	0	28 (29)	57	47 (21–66)	NA	4 (4)	32 (30)
e7	Farb et al., 2005	CE-MRA (1.5)	0	28 (29)	57	47 (21–66)	NA	0 (0)	36 (34)
e8	Majoie et al., 2005	TOF-MRA (3.0)	0	20 (21)	55	49 (18–74)	100	0 (0)	21 (20)
e8	Majoie et al., 2005	ceTOF-MRA (3.0)	0	20 (21)	55	49 (18–74)	100	0 (0)	21 (20)
e9	Westerlaan et al., 2005	TOF-MRA (1.5)	4	27 (33)	87	52 (29–75)	84	2 (0)	31 (27)
e10	Pierot et al., 2006	TOF-MRA (1.5)	0	32 (42)	84	48* (25–73)	100	0 (0)	42 (32)
e10	Pierot et al., 2006	CE-MRA (1.5)	0	32 (42)	84	48* (25–73)	100	0 (0)	42 (32)
e11	Deutschmann et al., 2007	(ce)TOF-MRA (1.5)	0	127 (136)	70	50 (13–74)	NA	13 (13)	188 (188)
e12	Wong et al., 2007	TOF-MRA (1.5)	0	37 (42)	92	54 (34–79)	51	0 (0)	44 (39)
e13	Gauvrit et al., 2008	CE-MRA (1.5)	63	107 (107)	54	46* (14–77)	85	15 (15)	92 (92)
e14	Lubicz et al., 2008	CE-MRA (1.5)	0	55 (67)	67	46 (17–65)	46	0 (0)	67 (55)
e15	Ramgren et al., 2008	TOF-MRA (1.5)	0	37 (41)	59	51* (24–69)	95	0 (0)	41 (37)
e15	Ramgren et al., 2008	TOF-MRA (3.0)	0	37 (41)	59	51* (24–69)	95	0 (0)	41 (37)
e15	Ramgren et al., 2008	CE-MRA (3.0)	0	37 (41)	59	51* (24–69)	95	3 (0)	38 (34)
e16	Urbach et al., 2008	TOF-MRA (3.0)	0	50 (50)	68	47 (8–74)	100	0 (0)	50 (50)
e17	Wikstrom et al., 2008	TOF-MRA (1.5)	0	38 (45)	NA	NA (*NA*)	NA	0 (0)	47 (39)
e17	Wikstrom et al., 2008	ceTOF-MRA (1.5)	0	38 (45)	NA	NA (*NA*)	NA	0 (0)	47 (39)
e17	Wikstrom et al., 2008	CE-MRA (1.5)	0	38 (45)	NA	NA (*NA*)	NA	0 (0)	47 (39)
e18	Buhk et al., 2009	TOF-MRA (3.0)	0	22 (22)	36	48* (29–78)	NA	1 (1)	21 (21)
e19	Ferre et al., 2009	TOF-MRA (3.0)	0	51 (51)	49	51* (19–72)	100	1 (0)	50 (50)
e20	Kau et al., 2009	TOF-MRA (1.5)	0	32 (37)	59	52 (26–90)	78	0 (0)	37 (37)
e20	Kau et al., 2009	CE-MRA (1.5)	0	32 (37)	59	52 (26–90)	78	0 (0)	37 (37)

e21	Bakker et al., 2010	TOF-MRA (1.5)	49	141 (141)	67	54* (14–84)	86	0 (0)	141 (141)
e22	Kaufmann et al., 2010	TOF-MRA (1.5)	0	58 (63)	78	59 (38–77)	NA	0 (0)	63 (58)
e22	Kaufmann et al., 2010	TOF-MRA (3.0)	0	58 (63)	78	59 (38–77)	NA	0 (0)	63 (58)
e22	Kaufmann et al., 2010	CE-MRA (1.5)	1	57 (62)	78	59 (38–77)	NA	0 (0)	62 (57)
e22	Kaufmann et al., 2010	CE-MRA (3.0)	1	57 (62)	78	59 (38–77)	NA	0 (0)	62 (57)
e23	Schaafsma et al., 2010	TOF+CE-MRA (1.5+3.0)	106	311 (343)	70	51 (19–79)	85	5 (5)	381 (346)
e24	Nakiri et al., 2011	TOF-MRA (3.0)	0	30 (43)	77	55 (27–74)	NA	0 (0)	43 (30)
e24	Nakiri et al., 2011	CE-MRA (3.0)	0	30 (43)	77	55 (27–74)	NA	0 (0)	43 (30)
e25	Lavoie et al., 2012	TOF+CE-MRA (1.5)	125	149 (160)	72	53* (26–83)	62	0 (0)	167 (156)
e26	Pierot et al., 2012	TOF-MRA (3.0)	4	96 (125)	59	51 (25–75)	77	10 (0)	126 (104)
e26	Pierot et al., 2012	CE-MRA (3.0)	4	96 (125)	59	51 (25–75)	77	10 (0)	126 (104)
e27	Pierot et al., 2012	TOF-MRA (1.5)	4	96 (125)	59	51 (25–75)	77	10 (0)	126 (104)

* median

eTable 4C. Index Test (MRA)

Ref.	Authors, year	MRA sequence (and field strength in *Tesla*)	TR, *msec*	TE, *msec*	Flip angle, *degree*	Matrix size, *pixels*	Field of view, *mm×mm*	Slice orien-tation	Slices, *n*	Slice thick-ness, *mm*	Slice overlap, *mm*	Slab thick-ness, *mm*	Acquired voxel volume, mm^3
e1	Anzalone et al., 2000	TOF-MRA (1.5)	39–43	6.2–8.0	20	230×512 or 192×512	200×230 or 150×200	axial	NA	0.9	0.0	NA	0.34 or 0.27
e1	Anzalone et al., 2000	ceTOF-MRA (1.5)	39–43	6.2–8.0	20	230×512 or 192×512	200×230 or 150×200	axial	NA	0.9	0.0	NA	0.34 or 0.27
e2	Boulin et al., 2001	ceTOF-MRA (1.5)	26	2.4	30	256×512	180×240	axial	60	1.0	0.0	60	0.33
e3	Michardiere et al., 2001	TOF-MRA (1.5)	37	6.5	20	192×512	200×200	axial	62	1.2	0.0	74	0.49
e4	Cottier et al., 2003	TOF-MRA (1.5)	38	6.9	30	224×512	220×220	axial	46	1.2	0.0	55	0.51
e4	Cottier et al., 2003	ceTOF-MRA (1.5)	38	6.9	30	224×512	220×220	axial	46	1.2	0.0	55	0.51
e5	Okahara et al., 2004	TOF-MRA (1.5)	40	6.8	20	160×256	160×160	axial	NA	1.2	0.0	NA	0.75
e6	Yamada et al., 2004	TOF-MRA (1.5)	35	1.5–1.6	25	256×256	150×150	axial	60	0.6	0.0	36	0.22
e7	Farb et al., 2005	TOF-MRA (1.5)	33.3	3.0	30	256×192	220×165	axial	120	1.0	0.0	120	0.81
e7	Farb et al., 2005	CE-MRA (1.5)	6.2	1.7	30	320×320	220×165	coronal	76	1.0	0.0	76	0.35
e8	Majoie et al., 2005	TOF-MRA (3.0)	21	4	20	512×512	170×200	axial	160	1.0	NA	160	0.24
e8	Majoie et al., 2005	ceTOF-MRA (3.0)	21	4	20	512×512	170×200	axial	160	1.0	NA	160	0.24
e9	Westerlaan et al., 2005	TOF-MRA (1.5)	35	6.4	20	160×512	220×220	axial	24	1.5	0.0	36	0.89
e10	Pierot et al., 2006	TOF-MRA (1.5)	35	3.4	20	320×224	220×220	axial	NA	1.4	NA	NA	0.95
e10	Pierot et al., 2006	CE-MRA (1.5)	6.6	1.5	35	320×224	220×220	coronal	NA	1.4	0.0	NA	0.95
e11	Deutschmann et al., 2007	TOF-MRA (1.5)	28	6	20	400×250	200×200	axial	160	0.9	0.0	144	0.36
e11	Deutschmann et al., 2007	ceTOF-MRA (1.5)	28	6	20	400×250	200×200	axial	160	0.9	0.0	144	0.36
e12	Wong et al., 2007	TOF-MRA (1.5)	35	2.9	25	256×256	150×150	axial	NA	0.6	0.0	NA	0.21
e13	Gauvrit et al., 2008	CE-MRA (1.5)	6.8	2.3	35	150×512	250×250	coronal	40	1.5	0.0	60	1.2
e14	Lubicz et al., 2008	CE-MRA (1.5)	4.2	1.3	35	212×352	175×350	coronal	85	1.6	0.8	68	1.31
e15	Ramgren et al., 2008	TOF-MRA (1.5)	25	6.9	20	496×496	230×230	axial	100	1.0	0.5	50	0.21
e15	Ramgren et al., 2008	TOF-MRA (3.0)	26	3.5	20	640×367	250×250	axial	100	1.0	0.5	50	0.27
e15	Ramgren et al., 2008	CE-MRA (3.0)	5.8	2.0	30	368×329	250×250	coronal	100	0.5	0.0	50	0.52
e16	Urbach et al., 2008	TOF-MRA (3.0)	25	3.5	20	528×269	200×200	axial	150	1	0.0	150	0.28
e17	Wikstrom et al., 2008	TOF-MRA (1.5)	35	2.4	25	NA	NA	axial	NA	NA	NA	NA	0.56
e17	Wikstrom et al., 2008	ceTOF-MRA (1.5)	35	2.4	25	NA	NA	axial	NA	NA	NA	NA	0.56
e17	Wikstrom et al., 2008	CE-MRA (1.5)	6.3	2.2	35	NA	NA	coronal	NA	NA	0.0	NA	0.81
e18	Buhk et al., 2009	TOF-MRA (3.0)	28	4.6	18	448×282	200×157	axial	140	0.65	0.0	0.65	0.17

e19	Ferre et al., 2009	TOF-MRA (3.0)	18	3.4	20	354×279	210×189	axial	140	1.1	0.55	77	0.44
e20	Kau et al., 2009	TOF-MRA (1.5)	25	6.9	25	496×280	230×230	axial	175	1.0	0.5	87.5	0.36
e20	Kau et al., 2009	CE-MRA (1.5)	10	2.5	25	520×417	280×280	coronal	150	0.5	0.0	75	0.13
e21	Bakker et al., 2010	TOF-MRA (1.5)	38	5.1	25	384×640	230×256	axial	72	0.8	0.0	60	0.19
e22	Kaufmann et al., 2010	TOF-MRA (1.5)	36	6.9	25	256×224	180×180	axial	84	1.4	0.0	122	0.79
e22	Kaufmann et al., 2010	TOF-MRA (3.0)	38	3.4	25	512×256	180×180	axial	84	1.4	0.0	122	0.35
e22	Kaufmann et al., 2010	CE-MRA (1.5)	6.6	1.3	45	256×224	220×220	coronal	48	1.2	0.0	58	1.00
e22	Kaufmann et al., 2010	CE-MRA (3.0)	8.6	1.5	40	416×224	220×220	coronal	48	1.2	0.0	58	0.62
e23	Schaafsma et al. (a–c)*, 2010	TOF-MRA (1.5)	25	6.9	20	496×496	200×200	axial	NA	1.0	0.5	NA	0.28
e23	Schaafsma et al. (d)*, 2010	TOF-MRA (1.5)	35	4.7	20	496×496	200×200	axial	NA	1.0	0.5	NA	0.28
e23	Schaafsma et al. (a–c)*, 2010	TOF-MRA (3.0)	20	4	20	512×512	200×170	axial	220	1.0	0.5	110	0.24
e23	Schaafsma et al. (a–c)*, 2010	CE-MRA (1.5)	6.2	1.9	30	400×400	210×180	coronal	NA	0.90	0.45	NA	0.32
e23	Schaafsma et al. (d)*, 2010	CE-MRA (1.5)	6.2	1.9	30	400×400	210×180	coronal	NA	0.90	0.45	NA	0.32
e23	Schaafsma et al. (a–c)*, 2010	CE-MRA (3.0)	5.3	1.7	30	368×368	250×200	coronal	NA	0.50	0.0	NA	0.52
e24	Nakiri et al., 2011	TOF-MRA (3.0)	25	3.5	20	528×270	200×200	axial	177	1.1	0.6	96	0.31
e24	Nakiri et al., 2011	CE-MRA (3.0)	5.6	2.1	27	244×244	220×220	coronal	220	1.2	0.6	130	0.97
e25	Lavoie et al., 2012	TOF-MRA (1.5)	23	7	25	204×256	165×210	axial	176	0.7	0.18	90	0.45
e25	Lavoie et al., 2012	CE-MRA (1.5)	3.2	1.2	30	187×384	150×192	coronal	96	1.0	-0.2	60	0.48
e26	Pierot et al., 2012	TOF-MRA (3.0)	18	3.45	20	464×418	210×190	axial	140	0.55	0.0	77	0.11
e26	Pierot et al., 2012	CE-MRA (3.0)	5.4	1.96	30	480×408	210×180	axial	110	0.50	0.0	55	0.10
e27	Pierot et al., 2012	TOF-MRA (1.5)	38	6.9	20	320×224	220×154	axial	110	0.7	0.0	77	0.33

NA, not available

* Multicenter study: (a) = Academic Medical Center Amsterdam; (b) = University Medical Center Leiden; (c) = University Medical Center Utrecht; (d) = Free University Medical Center Amsterdam.

eTable 4C. Index Test (MRA), *continued*

			Acquisition		Contrast medium				Image reading				
**Ref.**	**Authors, year**	**MRA sequence (and field strength in *Tesla*)**	**Parallel imaging factor**	**Acquisition time,** *min:sec*	**Generic name**	**Volume injected**	**Injection rate,** *mL/s*	**Method of bolus timing**	**Source images**	**MPR**	**MIP**	**VR**	**Observers** *n*
e1	Anzalone et al., 2000	TOF-MRA (1.5)	1	NA					+		+		1
e1	Anzalone et al., 2000	ceTOF-MRA (1.5)	1	NA	gadopentetate	0.1 mmol/kg	NA	NA	+		+		1
e2	Boulin et al., 2001	ceTOF-MRA (1.5)	1	5:23	gadopentetate	0.2 mmol/kg	NA	NA	+		+		1
e3	Michardiere et al., 2001	TOF-MRA (1.5)	1	7:22					+		+		NA
e4	Cottier et al., 2003	TOF-MRA (1.5)	1	4:57					+		+		2
e4	Cottier et al., 2003	ceTOF-MRA (1.5)	1	4:57	gadoterate	0.1 mmol/kg	manual	none	+		+		2
e5	Okahara et al., 2004	TOF-MRA (1.5)	1	10:02					+		+	+	2
e6	Yamada et al., 2004	TOF-MRA (1.5)	1	11:00					+		+		1
e7	Farb et al., 2005	TOF-MRA (1.5)	1	8:30					+		+		3
e7	Farb et al., 2005	CE-MRA (1.5)	1	2:00	gadodiamide	30 mL	3.0	fluoroscopy	+		+		3
e8	Majoie et al., 2005	TOF-MRA (3.0)	1.5	7:14					+		+		2
e8	Majoie et al., 2005	ceTOF-MRA (3.0)	1.5	7:14	gadopentetate	0.2 mmol/kg	NA	NA	+		+		2
e9	Westerlaan et al., 2005	TOF-MRA (1.5)	1	6:44					+		+		1
e10	Pierot et al., 2006	TOF-MRA (1.5)	1	6:00					+		+		2
e10	Pierot et al., 2006	CE-MRA (1.5)	1	0:58	gadoterate	10 mmol	2.0	fluoroscopy	+		+		2
e11	Deutschmann et al., 2007	TOF-MRA (1.5)	1	NA					+		+		2
e11	Deutschmann et al., 2007	ceTOF-MRA (1.5)	1	NA	gadodiamide	0.2 mmol/kg	NA	NA	+		+		2
e12	Wong et al., 2007	TOF-MRA (1.5)	1	9:08					+		+	+	2
e13	Gauvrit et al., 2008	CE-MRA (1.5)	1	0:40	gadodiamide	0.2 mmol/kg	2.0	test bolus	+		+		2
e14	Lubicz et al., 2008	CE-MRA (1.5)	1	0:49	gadobenate	9 mmol	1.8	fluoroscopy	+		+		2
e15	Ramgren et al., 2008	TOF-MRA (1.5)	1.5	7:38					+	+	+	+	3
e15	Ramgren et al., 2008	TOF-MRA (3.0)	2.0	5:58					+	+	+	+	3
e15	Ramgren et al., 2008	CE-MRA (3.0)	1.4	0:49	gadopentetate	9 mmol	2.0	test bolus	+	+	+	+	3
e16	Urbach et al., 2008	TOF-MRA (3.0)	2.0	NA					+		+		2
e17	Wikstrom et al., 2008	TOF-MRA (1.5)	1	5:00					+	+	+	+	1
e17	Wikstrom et al., 2008	ceTOF-MRA (1.5)	1	5:00	gadodiamide	40 mL	2.0	fluoroscopy	+	+	+	+	1
e17	Wikstrom et al., 2008	CE-MRA (1.5)	1	0:37	gadodiamide	40 mL	2.0	fluoroscopy	+	+	+	+	1
e18	Buhk et al., 2009	TOF-MRA (3.0)	1	5:12					+		+	+	3
e19	Ferre et al., 2009	TOF-MRA (3.0)	2.0	4:59					+		+	+	2
e20	Kau et al., 2009	TOF-MRA (1.5)	2.0	4:36					+		+		2
e20	Kau et al., 2009	CE-MRA (1.5)	2.0	3:00	gadofosveset	0.03 mmol/kg	manual	fixed delay	+		+		2
e21	Bakker et al., 2010	TOF-MRA (1.5)	1	5:30					+		+		3
e22	Kaufmann et al., 2010	TOF-MRA (1.5)	1	11:27					+	+	+		2

e22	Kaufmann et al., 2010	TOF-MRA (3.0)	1	12:06					+	+	+		2
e22	Kaufmann et al., 2010	CE-MRA (1.5)	1	0:51	gadodiamide	12.5 mmol	3.0	test bolus	+	+	+		2
e22	Kaufmann et al., 2010	CE-MRA (3.0)	1	0:51	gadodiamide	12.5 mmol	3.0	test bolus	+	+	+		2
e23	Schaafsma et al. (a–c)*, 2010	TOF-MRA (1.5)	2.0	6:00					+		+	+	2
e23	Schaafsma et al. (d)*, 2010	TOF-MRA (1.5)	2.0	6:00					+		+	+	2
e23	Schaafsma et al. (a–c)*, 2010	TOF-MRA (3.0)	1.5	7:00					+		+	+	2
e23	Schaafsma et al. (a–c)*, 2010	CE-MRA (1.5)	2.5	0:43	gadopentetate	15 mmol	3.0	test bolus	+		+	+	2
e23	Schaafsma et al. (d)*, 2010	CE-MRA (1.5)	2.5	0:43	gadopentetate	15 mmol	3.0	test bolus	+		+	+	2
e23	Schaafsma et al. (a–c)*, 2010	CE-MRA (3.0)	2.0	0:36	gadopentetate	7.5 mmol	2.0	test bolus	+		+	+	2
e24	Nakiri et al., 2011	TOF-MRA (3.0)	3.0	7:05					+		+	+	2
e24	Nakiri et al., 2011	CE-MRA (3.0)	2.0	NA	gadobenate	0.1 mmol/kg	2.0	NA	+		+	+	2
e25	Lavoie et al., 2012	TOF-MRA (1.5)	1	4:00					+	+	+	+	1
e25	Lavoie et al., 2012	CE-MRA (1.5)	1	0:40	gadodiamide	0.2 mmol/kg	2.0	test bolus	+	+	+	+	1
e26	Pierot et al., 2012	TOF-MRA (3.0)	2.0	4:59					+		+		2
e26	Pierot et al., 2012	CE-MRA (3.0)	2.0	0:52	gadoterate	10 mmol	NA	fluoroscopy	+		+		2
e27	Pierot et al., 2012	TOF-MRA (1.5)	2.0	6:09					+		+		2

NA, not available

Image types: MPR = multiplanar reformation; MIP = maximum intensity projection; VR = volume rendering

eTable 4D. Reference Test (DSA)

Ref.	Authors, year	Contrast medium: Generic name	Contrast medium: Iodine content, *mg/mL*	Contrast medium: Volume per injection, *mL*	Catheter angiography: DSA projections	Catheter angiography: 3D-RA applied	Catheter angiography: Obser-vers
e1	Anzalone et al., 2000	NA	NA	NA	NA	No	1
e2	Boulin et al., 2001	iobitridol	300	8–10	multiple views	No	2
e3	Michardiere et al., 2001	NA	NA	NA	NA	No	NA
e4	Cottier et al., 2003	iobitridol	NA	8–10	selected views	No	1
e5	Okahara et al., 2004	iohexol	300	15–20 total	3D-RA	Yes	2
e6	Yamada et al., 2004	NA	NA	NA	AP, L, W	No	1
e7	Farb et al., 2005	iohexol	300	NA	AP, L	Yes	3
e8	Majoie et al., 2005	iodixanol	320	6–8	3 views including W	No	2
e9	Westerlaan et al., 2005	iohexol	300	8–12	standard and additional views	No	1
e10	Pierot et al., 2006	iomeprol	300	8	multiple views including W	No	1
e11	Deutschmann et al., 2007	iodixanol	270	8	AP, L, O	No	1
e12	Wong et al., 2007	NA	NA	NA	multiple views including W	Yes	2
e13	Gauvrit et al., 2008	NA	NA	NA	standard and additional views	No	1
e14	Lubicz et al., 2008	iodixanol	320	10	standard views	Yes	2
e15	Ramgren et al., 2008	iohexol	240	8–9	AP, L, W	No	3
e16	Urbach et al., 2008	iopamidol	300	5–7	standard views and W	No	2
e17	Wikstrom et al., 2008	iohexol	NA	NA	AP, L, O	No	1
e18	Buhk et al., 2009	iopromide	300	DSA: 8<br>3D-RA: 17	standard views	Yes	3
e19	Ferre et al., 2009	iobitridol	300	8–10	AP, L, W	No	2
e20	Kau et al., 2009	iodixanol	270	5–6	multiple views including W	No	1
e21	Bakker et al., 2010	iodixanol	270	8	AP, L, W	Yes	3
e22	Kaufmann et al., 2010	iohexol	300	DSA: 8–10<br>3D-RA: 16	multiple views	Yes	2
e23	Schaafsma et al., 2010	iodixanol	300	≤8	standard views and W	No	2
e24	Nakiri et al., 2011	iobitridol	300	8–10	AP, L, W	Yes	2
e25	Lavoie et al., 2012	iodixanol	270	8	AP, L, O, W	No	2
e26	Pierot et al., 2012	iodixanol	320	8–10	AP, L, W	No	2
e27	Pierot et al., 2012	iodixanol	320	8–10	AP, L, W	No	2

NA, not available

3D-RA, rotational angiography with three-dimensional tomographic reconstruction

DSA, digital subtraction angiography

DSA projections: AP = anterior-posterior; L = lateral; O = oblique; W = working view

eTable 5. Reasons for Exclusion of Patients and Aneurysms from the Primary Studies

eTable 5A. Exclusion of Patients from the Studies

Ref.	Authors, year	MRA sequence (and Field Strength in *Tesla*)	Patients excluded	Reasons for exclusion of patients from the study
e1	Anzalone et al., 2000	ceTOF-MRA (1.5)	25	25 patients did not receive ceTOF-MRA, whereas 24 non-selected patients underwent ceTOF-MRA in addition to TOF-MRA
e2	Boulin et al., 2001	ceTOF-MRA (1.5)	2	2 patients did not undergo MRA due to claustrophobia
e6	Yamada et al., 2004	TOF-MRA (1.5)	31	31 patients with their MRA not performed within 3 days after coiling (early phase) or within 7 days of follow-up DSA
e9	Westerlaan et al., 2005	TOF-MRA (1.5)	4	3 patients underwent MRA but not DSA due to advanced age or complications during previous DSA<br>1 patient underwent MRA but DSA was aborted because of an adverse reaction to contrast medium
e13	Gauvrit et al., 2008	CE-MRA (1.5)	63	13 patients with failure of embolization<br>11 patients because of early death<br>10 patients refused to undergo DS angiography<br>10 patients had major early recurrence of aneurysmal disease leading to retreatment<br>8 patients refused the follow-up<br>5 patients had their follow-up at another center<br>4 patients were lost to follow-up<br>2 patients had contraindication for MR angiography
e21	Bakker et al., 2010	TOF-MRA (1.5)	49	15 patients underwent MRA but not DSA due to severe disabilities, adverse reactions to contrast medium, or because they refused DSA<br>14 patients with symptomatic aneurysms did not receive DSA or MRA because of severe disabilities<br>10 patients with SAH did not receive DSA or MRA because they passed away within 3 months after coiling, all related to initial SAH<br>10 patients underwent DSA but not MRA due to claustrophobia, implants, previous neurosurgical clipping, or additional aneurysms
e22	Kaufmann et al., 2010	CE-MRA (1.5)	1	1 patient could not undergo both follow-up conventional angiography and CE-MRA within 10 days
e22	Kaufmann et al., 2010	CE-MRA (3.0)	1	1 patient could not undergo both follow-up conventional angiography and CE-MRA within 10 days
e23	Schaafsma et al., 2010	TOF-MRA, CE-MRA (1.5, 3.0)	106	25 patients because of no informed consent<br>24 patients with neurosurgical clip<br>21 patients due to logistic problems<br>21 patients with reasons unknown<br>15 patients did not undergo MRA due to claustrophobia
e25	Lavoie et al., 2012	TOF-MRA, CE-MRA (1.5)	125	108 patients randomly not included since the MRA could not be performed on the day of DSA for logistic reasons<br>8 patients with MRA source images lost<br>4 patients with with MRA cancelled due to agition

e26	Pierot et al., 2012	TOF-MRA, CE-MRA (3.0)	4	3 patients with refusal to perform MRA<br>1 patient with non-MRA compatible clip<br>1 patient with aneurysm previously treated with clip<br>1 patient treated by parent vessel occlusion<br>2 patients treated with coiling and stent placement<br>1 patient who refused gadolinium injection
e27	Pierot et al., 2012	TOF-MRA (1.5, 3.0)	4	1 patient treated by parent vessel occlusion<br>2 patients treated with coiling and stent placement<br>1 patient in whom the 1.5 Tesla MRA images were not retrievable

eTable 5B. Exclusion of MRAs and Aneurysms from Analysis

Ref.	Authors, year	MRA sequence (and Field Strength in *Tesla*)	MRAs_(and aneurysms) excluded	Reasons for exclusion of MRAs and aneurysms from analysis
e1	Anzalone et al., 2000	TOF-MRA (1.5)	7 (7)	7 MRA with significant artifacts in association with the coils
e1	Anzalone et al., 2000	ceTOF-MRA (1.5)	4 (4)	4 MRA with coil-related artifacts in aneurysms that were occluded at DSA
e2	Boulin et al., 2001	ceTOF-MRA (1.5)	1 (1)	1 MRA with susceptibility artifacts from a nearby clipped aneurysm
e4	Cottier et al., 2003	TOF-MRA (1.5)	1 (1)	1 MRA with artifacts due to a neurosurgical clip
e4	Cottier et al., 2003	ceTOF-MRA (1.5)	1 (1)	1 MRA with artifacts due to a neurosurgical clip
e5	Okahara et al., 2004	TOF-MRA (1.5)	3 (3)	3 MRA with susceptibility artifacts at the aneurysm's neck site
e7	Farb et al., 2005	TOF-MRA (1.5)	4 (4)	4 MRA with imaging failures
e9	Westerlaan et al., 2005	TOF-MRA (1.5)	0 (2)	2 MRA (each with 3 aneurysms) were analyzed, but one aneurysm of each MRA was excluded because of susceptibility artifacts from nearby clipped aneurysms
e11	Deutschmann et al., 2007	TOF-MRA, CE-MRA (1.5)	13 (13)	8 MRA with interval between DSA and MRA more than 3 days<br>1 MRA with susceptibility artifacts due to a ventricular shunt<br>1 MRA with susceptibility artifacts due to surgical clips<br>1 MRA of a giant aneurysm with flow artifacts due to turbulences<br>2 MRA due to motion artifacts in addition to other artifacts
e13	Gauvrit et al., 2008	CE-MRA (1.5)	15 (15)	15 MRA with lack or failure of early or late contrast-enhancement
e15	Ramgren et al., 2008	CE-MRA (3.0)	3 (3)	3 MRA were not evaluable because of bad contrast timing
e18	Buhk et al., 2009	TOF-MRA (3.0)	1 (1)	1 MRA because of clip-related artifacts
e19	Ferre et al., 2009	TOF-MRA (3.0)	1 (1)	1 MRA because the corresponding DSA was not interpretable
e23	Schaafsma et al., 2010	TOF-MRA, CE-MRA (1.5, 3.0)	5 (5)	1 MRA was discontinued and thus not interpretable<br>1 MRA was not interpretable<br>3 MRAs because the correlated DSA was not interpretable
e25	Lavoie et al., 2012	TOF-MRA (1.5)	8 (8)	4 MRA with coil artifacts<br>4 MRA with aneuysms not included in the field of view
e25	Lavoie et al., 2012	CE-MRA (1.5)	1 (1)	1 MRA with aneuysm not included in the field of view
e26	Pierot et al., 2012	TOF-MRA, CE-MRA (3.0)	0 (10)	9 MRA with multiple aneurysms were analyzed and 1 clipped aneurysm was excluded<br>1 MRA with multiple aneurysms were analyzed and 1 stented aneurysm was excluded
e27	Pierot et al., 2012	TOF-MRA (1.5, 3.0)	0 (10)	9 MRA with multiple aneurysms were analyzed and 1 clipped aneurysm was excluded<br>1 MRA with multiple aneurysms were analyzed and 1 stented aneurysm was excluded

eTable 6. Bivariate Count Data of MRA versus DSA

eTable 6A. Bivariate Assessment of Any Residuals

			DSA: no residual		*DSA:* neck or sac residual				
			MRA: no residual,	*MRA:* neck or sac residual,	*MRA:* no residual,	*MRA:* neck or sac residual,		*Diagnosing Any Residuals:*	
**Ref.**	**Authors, year**	**MRA Sequence (and Field Strength in *Tesla*)**	***n***	***n***	***n***	***n***	**sum, *n***	**Sensitivity, mean (95%-CI), %**	**Specificity, mean (95%-CI), %**
e1	Anzalone et al., 2000	TOF-MRA (1.5)	38	1	0	18	57	100 (81.5–100)	97.4 (86.5–99.9)
e1	Anzalone et al., 2000	ceTOF-MRA (1.5)	13	1	0	7	21	100 (59.0–100)	92.9 (66.1–99.8)
e2	Boulin et al., 2001	ceTOF-MRA (1.5)	54	1	7	18	80	72.0 (50.6–87.9)	98.2 (90.3–100)
e3	Michardiere et al., 2001	TOF-MRA (1.5)	9	0	4	12	25	75.0 (47.6–92.7)	100 (66.4–100)
e4	Cottier et al., 2003	TOF-MRA (1.5)	34	0	6	30	70	83.3 (67.2–93.6)	100 (89.7–100)
e4	Cottier et al., 2003	ceTOF-MRA (1.5)	34	0	6	30	70	83.3 (67.2–93.6)	100 (89.7–100)
e5	Okahara et al., 2004	TOF-MRA (1.5)	10	0	4	16	30	80.0 (56.3–94.3)	100 (69.2–100)
e6	Yamada et al., 2004	TOF-MRA (1.5)	13	13	0	25	51	100 (86.3–100)	50.0 (29.9–70.1)
e7	Farb et al., 2005	TOF-MRA (1.5)	20	2	6	4	32	40.0 (12.2–73.8)	90.9 (70.8–98.9)
e7	Farb et al., 2005	CE-MRA (1.5)	22	3	2	9	36	81.8 (48.2–97.7)	88.0 (68.8–97.5)
e8	Majoie et al., 2005	TOF-MRA (3.0)	9	3	0	9	21	100 (66.4–100)	75.0 (42.8–94.5)
e8	Majoie et al., 2005	ceTOF-MRA (3.0)	9	3	0	9	21	100 (66.4–100)	75.0 (42.8–94.5)
e9	Westerlaan et al., 2005	TOF-MRA (1.5)	20	2	1	8	31	88.9 (51.8–99.7)	90.9 (70.8–98.9)
e10	Pierot et al., 2006 [&]	TOF-MRA (1.5)	13	1	5.5	22.5	42	80.4 (61.1–92.8)	92.9 (66.1–99.8)
e10	Pierot et al., 2006 [&]	CE-MRA (1.5)	12.5	1.5	6	22	42	78.6 (59.0–91.7)	89.3 (61.5–99.2)
e11	Deutschmann et al., 2007	TOF-MRA (1.5)	118	9	7	54	188	88.5 (77.8–95.3)	92.9 (87.0–96.7)
e12	Wong et al., 2007	TOF-MRA (1.5)	9	2	0	33	44	100 (89.4–100)	81.8 (48.2–97.7)
e13	Gauvrit et al., 2008	CE-MRA (1.5)	60	0	1	31	92	96.9 (83.8–99.9)	100 (94.0–100)
e14	Lubicz et al., 2008 [&]	CE-MRA (1.5)	50.5	2.5	1	13	67	92.9 (66.1–99.8)	95.3 (85.7–99.2)
e15	Ramgren et al., 2008	TOF-MRA (1.5)	2	4	4	31	41	88.6 (73.3–96.8)	33.3 (4.3–77.7)
e15	Ramgren et al., 2008	TOF-MRA (3.0)	4	2	3	32	41	91.4 (76.9–98.2)	66.7 (22.3–95.7)
e15	Ramgren et al., 2008	CE-MRA (3.0)	2	3	6	27	38	81.8 (64.5–93.0)	40.0 (5.3–85.3)
e16	Urbach et al., 2008	TOF-MRA (3.0)	36	3	0	11	50	100 (71.5–100)	92.3 (79.1–98.4)
e17	Wikstrom et al., 2008	TOF-MRA (1.5)	18	7	1	21	47	95.5 (77.2–99.9)	72.0 (50.6–87.9)
e17	Wikstrom et al., 2008	ceTOF-MRA (1.5)	17	8	1	21	47	95.5 (77.2–99.9)	68.0 (46.5–85.1)

e17	Wikstrom et al., 2008	CE-MRA (1.5)	19	6	4	18	47	81.8 (59.7–94.8)	76.0 (54.9–90.6)
e18	Buhk et al., 2009 §	TOF-MRA (3.0)	11	2.67	0.33	7	21	95.5 (54.4–100)	80.5 (50.8–96.3)
e19	Ferre et al., 2009	TOF-MRA (3.0)	23	3	0	24	50	100 (85.8–100)	88.5 (69.8–97.6)
e20	Kau et al., 2009	TOF-MRA (1.5)	10	3	3	21	37	87.5 (67.6–097.3)	76.9 (46.2–95.0)
e20	Kau et al., 2009	CE-MRA (1.5)	12	1	2	22	37	91.7 (73.0–99.0)	92.3 (64.0–99.8)
e21	Bakker et al., 2010	TOF-MRA (1.5)	128	0	2	11	141	84.6 (54.6–98.1)	100 (97.2–100)
e22	Kaufmann et al., 2010	TOF-MRA (1.5)	12	11	4	36	63	90.0 (76.3–97.2)	52.2 (30.6–73.2)
e22	Kaufmann et al., 2010	TOF-MRA (3.0)	12	11	5	35	63	87.5 (73.2–95.8)	52.2 (30.6–73.2)
e22	Kaufmann et al., 2010	CE-MRA (1.5)	15	8	6	33	62	84.6 (69.5–94.1)	65.2 (42.7–83.6)
e22	Kaufmann et al., 2010	CE-MRA (3.0)	14	8	4	36	62	90.0 (76.3–97.2)	63.6 (40.7–0.828)
e23	Schaafsma et al. (a)*, 2010	TOF+CE-MRA (1.5+3.0)	20	15	5	39	79	88.6 (75.4–96.2)	57.1 (39.4–0.737)
e23	Schaafsma et al. (b)*, 2010	TOF+CE-MRA (1.5+3.0)	27	13	2	35	77	94.6 (81.8–99.3)	67.5 (50.9–0.814)
e23	Schaafsma et al. (c)*, 2010	TOF+CE-MRA (1.5+3.0)	58	43	8	55	164	87.3 (76.5–94.4)	57.4 (47.2–0.672)
e23	Schaafsma et al. (d)*, 2010	TOF+CE-MRA (1.5)	18	5	4	34	61	89.5 (75.2–97.1)	78.3 (56.3–0.925)
e24	Nakiri et al., 2011	TOF-MRA (3.0)	23	0	1	19	43	95.0 (75.1–99.9)	100 (85.2–100)
e24	Nakiri et al., 2011	CE-MRA (3.0)	23	0	0	20	43	100 (83.2–100)	100 (85.2–100)
e25	Lavoie et al., 2012	TOF-MRA (1.5)	50	12	21	76	159	78.4 (68.8–86.1)	80.6 (68.6–0.896)
e25	Lavoie et al., 2012	CE-MRA (1.5)	53	14	12	87	166	87.9 (79.8–93.6)	79.1 (67.4–0.881)
e26	Pierot et al., 2012	TOF-MRA (3.0)	47	10	18	51	126	73.9 (61.9–83.7)	82.5 (70.1–0.913)
e26	Pierot et al., 2012	CE-MRA (3.0)	49	8	19	50	126	72.5 (60.4–82.5)	86.0 (74.2–0.937)
e27	Pierot et al., 2012	TOF-MRA (1.5)	51	7	24	44	126	64.7 (52.2–75.9)	87.9 (76.7–0.950)

Symbols:

& Count data were averaged from 2 observers.

§ Count data were averaged from 3 observers.

* Multicenter study: (a) = Academic Medical Center Amsterdam; (b) = University Medical Center Leiden; (c) = University Medical Center Utrecht; (d) = Free University Medical Center Amsterdam.

Abbreviations:

95%-CI, 95% confidence interval
DSA, digital subtraction angiography
MRA, magnetic resonance angiography:
TOF-MRA, time-of-flight MRA
ceTOF-MRA, contrast-enhanced TOF-MRA
CE-MRA, contrast-enhanced T1-weighted MRA

eTable 6B. Bivariate Assessment of Sac Residuals

Ref.	Authors, year	MRA Sequence (and Field Strength in *Tesla*)	*DSA:* no or neck residual — *MRA:* no residual, *n*	*DSA:* no or neck residual — *MRA:* neck or sac residual, *n*	*DSA:* sac residual — *MRA:* no residual, *n*	*DSA:* sac residual — *MRA:* neck or sac residual, *n*	sum, *n*	*Diagnosing Sac Residuals:* Sensitivity, mean (95%-CI), %	*Diagnosing Sac Residuals:* Specificity, mean (95%-CI), %
e1	Anzalone et al., 2000	TOF-MRA (1.5)	55	0	0	2	57	100 (15.8–100)	100 (93.5–100)
e3	Michardiere et al., 2001	TOF-MRA (1.5)	21	0	1	3	25	75.0 (19.4–99.4)	100 (83.9–100)
e5	Okahara et al., 2004	TOF-MRA (1.5)	27	0	0	3	30	100 (29.2–100)	100 (87.2–100)
e6	Yamada et al., 2004	TOF-MRA (1.5)	49	1	0	1	51	100 (2.5–100)	98.0 (89.4–99.9)
e9	Westerlaan et al., 2005	TOF-MRA (1.5)	30	1	0	0	31	ND	96.8 (83.3–99.9)
e10	Pierot et al., 2006 [&]	TOF-MRA (1.5)	36.5	0.5	0.5	4.5	42	90.0 (37.1–100)	98.6 (88.1–100)
e10	Pierot et al., 2006 [&]	CE-MRA (1.5)	36.5	0.5	0.5	4.5	42	90.0 (37.1–100)	98.6 (88.1–100)
e12	Wong et al., 2007	TOF-MRA (1.5)	23	2	0	19	44	100 (82.4–100)	92.0 (74.0–99.0)
e13	Gauvrit et al., 2008	CE-MRA (1.5)	80	0	2	10	92	83.3 (51.6–97.9)	100 (95.5–100)
e14	Lubicz et al., 2008 [&]	CE-MRA (1.5)	60	0	1	6	67	85.7 (42.1–99.6)	100 (94.0–100)
e15	Ramgren et al., 2008	TOF-MRA (1.5)	28	5	3	5	41	62.5 (24.5–91.5)	84.8 (68.1–94.9)
e15	Ramgren et al., 2008	TOF-MRA (3.0)	33	2	2	4	41	66.7 (22.3–95.7)	94.3 (80.8–99.3)
e15	Ramgren et al., 2008	CE-MRA (3.0)	27	4	2	5	38	71.4 (29.0–96.3)	87.1 (70.2–96.4)
e16	Urbach et al., 2008	TOF-MRA (3.0)	41	0	0	9	50	100 (66.4–100)	100 (91.4–100)
e18	Buhk et al., 2009 [§]	TOF-MRA (3.0)	16.67	0.33	0	4	21	100 (39.8–100)	98.1 (77.2–100)
e19	Ferre et al., 2009	TOF-MRA (3.0)	44	2	0	4	50	100 (39.8–100)	95.7 (85.2–99.5)
e20	Kau et al., 2009	TOF-MRA (1.5)	23	3	2	9	37	81.8 (48.2–97.7)	88.5 (69.8–97.6)
e20	Kau et al., 2009	CE-MRA (1.5)	23	3	3	8	37	72.7 (39.0–94.0)	88.5 (69.8–97.6)
e21	Bakker et al., 2010	TOF-MRA (1.5)	136	0	0	5	141	100 (47.8–100)	100 (97.3–100)
e22	Kaufmann et al., 2010	TOF-MRA (1.5)	48	7	2	6	63	75.0 (34.9–96.8)	87.3 (75.5–94.7)
e22	Kaufmann et al., 2010	TOF-MRA (3.0)	45	10	2	6	63	75.0 (34.9–96.8)	81.8 (69.1–90.9)
e22	Kaufmann et al., 2010	CE-MRA (1.5)	43	11	1	7	62	87.5 (47.3–99.7)	79.6 (66.5–89.4)
e22	Kaufmann et al., 2010	CE-MRA (3.0)	45	9	1	7	62	87.5 (47.3–99.7)	83.3 (70.7–92.1)
e23	Schaafsma et al. (a)*, 2010	TOF+CE-MRA (1.5+3.0)	65	1	3	10	79	76.9 (46.2–95.0)	98.5 (91.8–100)
e23	Schaafsma et al. (b)*, 2010	TOF+CE-MRA (1.5+3.0)	47	5	5	20	77	80.0 (59.3–93.2)	90.4 (79.0–96.8)
e23	Schaafsma et al. (c)*, 2010	TOF+CE-MRA (1.5+3.0)	115	16	5	28	164	84.8 (68.1–94.9)	87.8 (80.9–92.9)
e23	Schaafsma et al. (d)*, 2010	TOF+CE-MRA (1.5)	34	10	3	14	61	82.4 (56.6–96.2)	77.3 (62.2–88.5)
e24	Nakiri et al., 2011	TOF-MRA (3.0)	29	0	0	14	43	100 (76.8–100)	100 (88.1–100)
e24	Nakiri et al., 2011	CE-MRA (3.0)	29	0	0	14	43	100 (76.8–100)	100 (88.1–100)
e25	Lavoie et al., 2012	TOF-MRA (1.5)	97	17	11	34	159	75.6 (60.5–87.1)	85.1 (77.2–91.1)

e25	Lavoie et al., 2012	CE-MRA (1.5)	103	18	9	36	166	80.0 (65.4–90.4)	85.1 (77.5–90.9)
e26	Pierot et al., 2012	TOF-MRA (3.0)	85	6	9	26	126	74.3 (56.7–87.5)	93.4 (86.2–97.5)
e26	Pierot et al., 2012	CE-MRA (3.0)	83	8	7	28	126	80.0 (63.1–91.6)	91.2 (83.4–96.1)
e27	Pierot et al., 2012	TOF-MRA (1.5)	89	2	16	19	126	54.3 (36.6–71.2)	97.8 (92.3–99.7)

Symbols:

& Count data were averaged from 2 observers.

§ Count data were averaged from 3 observers.

* Multicenter study: (a) = Academic Medical Center Amsterdam; (b) = University Medical Center Leiden; (c) = University Medical Center Utrecht; (d) = Free University Medical Center Amsterdam.

Abbreviations:

95%-CI, 95% confidence interval
DSA, digital subtraction angiography
MRA, magnetic resonance angiography:
  TOF-MRA, time-of-flight MRA
  ceTOF-MRA, contrast-enhanced TOF-MRA
  CE-MRA, contrast-enhanced T1-weighted MRA
ND, not determined

eTable 7. Trivariate Count Data of MRA versus DSA

			DSA: no residual			*DSA:* neck residual			*DSA:* sac residual			
**Ref.**	**Authors, year**	**MRA Sequence (and Field Strength in *Tesla*)**	***MRA:* no residual, *n***	***MRA:* neck residual, *n***	***MRA:* sac residual, *n***	***MRA:* no residual, *n***	***MRA:* neck residual, *n***	***MRA:* sac residual, *n***	***MRA:* no residual, *n***	***MRA:* neck residual, *n***	***MRA:* sac residual, *n***	**sum, *n***
e1	Anzalone et al., 2000	TOF-MRA (1.5)	38	1	0	0	16	0	0	0	2	57
e3	Michardiere et al., 2001	TOF-MRA (1.5)	9	0	0	3	9	0	1	0	3	25
e5	Okahara et al., 2004	TOF-MRA (1.5)	10	0	0	4	13	0	0	0	3	30
e6	Yamada et al., 2004	TOF-MRA (1.5)	13	12	1	0	24	0	0	0	1	51
e9	Westerlaan et al., 2005	TOF-MRA (1.5)	20	1	1	1	8	0	0	0	0	31
e10	Pierot et al., 2006 [&]	TOF-MRA (1.5)	13	1	0	5.5	17	0.5	0	0.5	4.5	42
e10	Pierot et al., 2006 [&]	CE-MRA (1.5)	12.5	1.5	0	6	16.5	0.5	0	0.5	4.5	42
e12	Wong et al., 2007	TOF-MRA (1.5)	9	2	0	0	12	2	0	0	19	44
e13	Gauvrit et al., 2008	CE-MRA (1.5)	60	0	0	1	19	0	0	2	10	92
e14	Lubicz et al., 2008 [&]	CE-MRA (1.5)	50.5	2.5	0	0	7	0	1	0	6	67
e15	Ramgren et al., 2008	TOF-MRA (1.5)	2	4	0	3	19	5	1	2	5	41
e15	Ramgren et al., 2008	TOF-MRA (3.0)	4	2	0	2	25	2	1	1	4	41
e15	Ramgren et al., 2008	CE-MRA (3.0)	2	3	0	5	17	4	1	1	5	38
e16	Urbach et al., 2008	TOF-MRA (3.0)	36	3	0	0	2	0	0	0	9	50
e18	Buhk et al., 2009 [§]	TOF-MRA (3.0)	11	2.67	0	0.33	2.67	0.33	0	0	4	21
e19	Ferre et al., 2009	TOF-MRA (3.0)	23	2	1	0	19	1	0	0	4	50
e20	Kau et al., 2009	TOF-MRA (1.5)	10	3	0	3	7	3	0	2	9	37
e20	Kau et al., 2009	CE-MRA (1.5)	12	1	0	2	8	3	0	3	8	37
e21	Bakker et al., 2010	TOF-MRA (1.5)	128	0	0	2	6	0	0	0	5	141
e22	Kaufmann et al., 2010	TOF-MRA (1.5)	12	8	3	4	24	4	0	2	6	63
e22	Kaufmann et al., 2010	CE-MRA (1.5)	15	4	4	6	18	7	0	1	7	62
e22	Kaufmann et al., 2010	TOF-MRA (3.0)	12	6	5	5	22	5	0	2	6	63
e22	Kaufmann et al., 2010	CE-MRA (3.0)	14	5	3	4	22	6	0	1	7	62
e23	Schaafsma et al. (a)*,2010	TOF+CE-MRA (1.5+3.0)	20	15	0	5	25	1	0	3	10	79
e23	Schaafsma et al. (b)*,2010	TOF+CE-MRA (1.5+3.0)	27	11	2	2	7	3	0	5	20	77
e23	Schaafsma et al. (c)*,2010	TOF+CE-MRA (1.5+3.0)	58	34	9	7	16	7	1	4	28	164
e23	Schaafsma et al. (d)*,2010	TOF+CE-MRA (1.5)	18	4	1	4	8	9	0	3	14	61
e24	Nakiri et al., 2011	TOF-MRA (3.0)	23	0	0	1	5	0	0	0	14	43
e24	Nakiri et al., 2011	CE-MRA (3.0)	23	0	0	0	6	0	0	0	14	43
e25	Lavoie et al., 2012	TOF-MRA (1.5)	50	7	5	18	22	12	3	8	34	159
e25	Lavoie et al., 2012	CE-MRA (1.5)	53	9	5	12	29	13	0	9	36	166
e26	Pierot et al., 2012	TOF-MRA (3.0)	47	8	2	15	15	4	3	6	26	126

e26	Pierot et al., 2012	CE-MRA (3.0)	49	7	1	15	12	7	4	3	28	126
e27	Pierot et al., 2012	TOF-MRA (1.5)	51	7	0	16	15	2	8	8	19	126

Symbols:

[&] Count data were averaged from 2 observers.

[§] Count data were averaged from 3 observers.

* Multicenter study: (a) = Academic Medical Center Amsterdam; (b) = University Medical Center Leiden; (c) = University Medical Center Utrecht; (d) = Free University Medical Center Amsterdam.

Abbreviations:

DSA, digital subtraction angiography
MRA, magnetic resonance angiography:
TOF-MRA, time-of-flight MRA
CE-MRA, contrast-enhanced T1-weighted MRA

eTable 8. Subgroup Analyses with Study Characteristics as Covariates

Covariates	*Number of subgroups*	LOR* estimate, *(95%-CI)*	*P*-value
**Study quality score**			
**A.** (10.5–12.0)	4	6.86 (5.13–9.45)	0.071
**B.** (12.5–14.0)	20	5.11 (4.10–6.37)	
**Prospective study**			
**A.** (no)	5	5.97 (3.63–8.47)	0.535
**B.** (yes)	18	5.17 (4.09–6.53)	
**Patients per study**			
**A.** (< 50 patients)	12	6.37 (4.84–8.18)	0.132
**B.** (≥ 50 patients)	12	4.84 (3.58–6.34)	
**Percentage of women**			
**A.** (< 70%)	13	6.15 (4.71–7.94)	0.209
**B.** (≥ 70%)	11	4.88 (3.49–6.49)	
**Mean or median age**			
**A.** (≤ 51 years)	14	5.18 (3.85–6.74)	0.300
**B.** (> 51 years)	10	6.35 (4.58–8.51)	
**Ruptured aneurysms**			
**A.** (<84.9% of patients)	13	4.91 (3.46–6.62)	0.584
**B.** (≥ 84.9% of patients)	11	5.46 (4.11–7.10)	
**MRA sequence type**			
**A.** (TOF-MRA)	21	5.27 (4.17–6.60)	0.566
**B.** (CE-MRA)	10	4.73 (3.24–6.37)	
**MR field strength**			
**A.** (1.5 Tesla)	20	4.98 (3.91–6.25)	0.998
**B.** (3.0 Tesla)	12	4.97 (3.63–6.52)	
**MR: Parallel imaging**			
**A.** (No)	18	5.61 (4.38–7.08)	0.259
**B.** (Yes)	13	4.60 (3.29–6.08)	
**MR: Voxel volume**			
**A.** (≤ 0.35 mm^3)	19	5.14 (3.89–6.65)	0.957
**B.** (> 0.35 mm^3)	16	5.09 (3.86–6.52)	
**MRA image readers**			
**A.** (1 reader)	5	4.87 (2.90–7.17)	0.965
**B.** (2–3 readers)	29	4.80 (3.99–5.76)	
**DSA iodine concent**			
**A.** (240–270 mg/mL)	9	4.47 (2.08–7.15)	0.498
**B.** (300–320 mg/mL)	15	5.38 (4.07–7.08)	
**Rotational angiography**			
**A.** (no)	17	5.97 (3.63–8.47)	0.535
**B.** (yes)	7	5.17 (4.09–6.53)	

**DSA image readers**			
**A.** (1 reader)	6	6.32 (4.07−8.82)	0.388
**B.** (2−3 readers)	17	5.21 (4.06−6.67)	

* LOR, logarithm of the diagnostic odds ratio

These subgroup analyses were performed with bivariate random-effects meta-analyses of sensitivity and specificity for the assessment of "sac residuals" with the study characteristics as covariates. The according differences of the LOR (logarithm of the diagnostic odd ratio) showed some trends that were not significant ($P > 0.05$). The two-sided P-values were determined from the percentiles of the according posterior distributions.

eTable 9. Supplemental References of the Primary Studies

e1. Anzalone N, Righi C, Simionato F, et al (2000) Three-dimensional time-of-flight MR angiography in the evaluation of intracranial aneurysms treated with Guglielmi detachable coils. Am J Neuroradiol 21:746–752.
e2. Boulin A, Pierot L (2001) Follow-up of intracranial aneurysms treated with detachable coils: comparison of gadolinium-enhanced 3D time-of-flight MR angiography and digital subtraction angiography. Radiology 219:108–113.
e3. Michardière R, Bensalem D, Martin D, Baudouin N, Binnert D (2001) Comparaison de l'ARM et de l'artériographie dans le suivi des anévrismes intracrâniens traités par GDC. J Neuroradiol 28:75–83.
e4. Cottier JP, Bleuzen-Couthon A, Gallas S, et al (2003) Intracranial aneurysms treated with Guglielmi detachable coils: is contrast material necessary in the follow-up with 3D time-of-flight MR angiography? Am J Neuroradiol 24:1797–1803.
e5. Okahara M, Kiyosue H, Hori Y, Yamashita M, Nagatomi H, Mori H (2004) Three-dimensional time-of-flight MR angiography for evaluation of intracranial aneurysms after endosaccular packing with Guglielmi detachable coils: comparison with 3D digital subtraction angiography. Eur Radiol 14:1162–1168.
e6. Yamada N, Hayashi K, Murao K, Higashi M, Iihara K (2004) Time-of-flight MR angiography targeted to coiled intracranial aneurysms is more sensitive to residual flow than is digital subtraction angiography. Am J Neuroradiol 25:1154–1157.
e7. Farb RI, Nag S, Scott JN, et al (2005) Surveillance of intracranial aneurysms treated with detachable coils: a comparison of MRA techniques. Neuroradiology 47:507–515.
e8. Majoie CBLM, Sprengers ME, van Rooij WJJ, et al (2005) MR angiography at 3T versus digital subtraction angiography in the follow-up of intracranial aneurysms treated with detachable coils. Am J Neuroradiol 26:1349–1356.
e9. Westerlaan HE, van der Vliet AM, Hew JM, et al (2005) Time-of-flight magnetic resonance angiography in the follow-up of intracranial aneurysms treated with Guglielmi detachable coils. Neuroradiology 47:622–629.
e10. Pierot L, Delcourt C, Bouquigny F, et al (2006) Follow-up of intracranial aneurysms selectively treated with coils: prospective evaluation of contrast-enhanced MR angiography. Am J Neuroradiol 27:744–749.
e11. Deutschmann HA, Augustin M, Simbrunner J, et al (2007) Diagnostic accuracy of 3D time-of-flight MR angiography compared with digital subtraction angiography for follow-up of coiled intracranial aneurysms: influence of aneurysm size. Am J Neuroradiol 28:628–634.
e12. Wong JH, Mitha AP, Willson M, Hudon ME, Sevick RJ, Frayne R (2007) Assessment of brain aneurysms by using high-resolution magnetic resonance angiography after endovascular coil delivery. J Neurosurg 107:283–289.
e13. Gauvrit JY, Caron S, Taschner CA, Lejeune JP, Pruvo JP, Leclerc X (2008) Intracranial aneurysms treated with Guglielmi detachable coils: long-term imaging follow-up with contrast-enhanced magnetic resonance angiography. J Neurosurg 108:443–449.
e14. Lubicz B, Neugroschl C, Collignon L, Francois O, Baleriaux D (2008) Is digital substraction angiography still needed for the follow-up of intracranial aneurysms treated by embolisation with detachable coils? Neuroradiology 50:841–848.
e15. Ramgren B, Siemund R, Cronqvist M, et al (2008) Follow-up of intracranial aneurysms treated with detachable coils: comparison of 3D inflow MRA at 3T and 1.5T and contrast-enhanced MRA at 3T with DSA. Neuroradiology 50:947–954.
e16. Urbach H, Dorenbeck U, von Falkenhausen M, et al (2008) Three-dimensional time-of-flight MR angiography at 3 T compared to digital subtraction angiography in the follow-up

of ruptured and coiled intracranial aneurysms: a prospective study. Neuroradiology 50:383–389.

e17. Wikström J, Ronne-Engström E, Gal G, Enblad P, Tovi M (2008) Three-dimensional time-of-flight (3D TOF) magnetic resonance angiography (MRA) and contrast-enhanced MRA of intracranial aneurysms treated with platinum coils. Acta Radiol 49:190−196.

e18. Buhk JH, Kallenberg K, Mohr A, Dechent P, Knauth M (2009) Evaluation of angiographic computed tomography in the follow-up after endovascular treatment of cerebral aneurysms – a comparative study with DSA and TOF-MRA. Eur Radiol 19:430−436.

e19. Ferré JC, Carsin-Nicol B, Morandi X, et al (2009) Time-of-flight MR angiography at 3T versus digital subtraction angiography in the imaging follow-up of 51 intracranial aneurysms treated with coils. Eur J Radiol 72:365–369.

e20. Kau T, Gasser J, Celedin S, et al (2009) MR angiographic follow-up of intracranial aneurysms treated with detachable coils: evaluation of a blood-pool contrast medium. Am J Neuroradiol 30:1524–1530.

e21. Bakker NA, Westerlaan HE, Metzemaekers JDM, et al (2010) Feasibility of magnetic resonance angiography (MRA) follow-up as the primary imaging modality after coiling of intracranial aneurysms. Acta Radiol 51:226–232.

e22. Kaufmann TJ, Huston J III, Cloft HJ, et al (2010) A prospective trial of 3T and 1.5T time-of-flight and contrast-enhanced MR angiography in the follow-up of coiled intracranial aneurysms. Am J Neuroradiol 31:912−918.

e23. Schaafsma JD, Velthuis BK, Majoie CBLM, et al (2010) Intracranial aneurysms treated with coil placement: test characteristics of follow-up MR angiography – multicenter study. Radiology 256:209–218.

e24. Nakiri GS, Santos AC, Abud TG, Aragon DC, Colli BO, Abud DG (2011) A comparison between magnetic resonance angiography at 3 teslas (time-of-flight and contrast-enhanced) and flat-panel digital subtraction angiography in the assessment of embolized brain aneurysms. Clinics (Sao Paulo) 66:641−648.

e25. Lavoie P, Gariépy JL, Milot G, et al (2012) Residual flow after cerebral aneurysm coil occlusion: diagnostic accuracy of MR angiography. Stroke 43:740−746.

e26. Pierot L, Portefaix C, Boulin A, Gauvrit JY (2012) Follow-up of coiled intracranial aneurysms: comparison of 3D time-of-flight and contrast-enhanced magnetic resonance angiography at 3T in a large, prospective series. Eur Radiol 22:2255–2263.

e27. Pierot L, Portefaix C, Gauvrit JY, Boulin A (2012) Follow-Up of Coiled Intracranial Aneurysms: Comparison of 3D Time-of-Flight MR Angiography at 3T and 1.5T in a Large Prospective Series. AJNR Am J Neuroradiol 33:2162–2166.
